# Molecular Modeling and *In Vitro* Evaluation of Thioureas and Arylthioureas as Urease Inhibitors

**DOI:** 10.1021/acsomega.5c01648

**Published:** 2025-05-22

**Authors:** Marciéli Fabris, Priscila G. Camargo, Mariana L. Silva, Camilo H. S. Lima, Magaly G. Albuquerque, Carlos R. Rodrigues, Nailton M. Nascimento-Júnior, Marcelle L. F. Bispo

**Affiliations:** † Laboratório de Síntese de Moléculas Medicinais (LaSMMed), Departamento de Química, Universidade Estadual de Londrina (UEL), Rodovia Celso Garcia Cid, PR-445, Km 380, Londrina, Paraná 86057-970, Brasil; ‡ Faculdade de Farmácia, Departamento de Fármacos e Medicamentos, 28125 Universidade Federal do Rio de Janeiro, Av. Carlos Chagas Filho, 373, Rio de Janeiro, Rio de Janeiro 21941-170, Brasil; § Laboratório de Modelagem Molecular (LabMMol), Instituto de Química, Universidade Federal do Rio de Janeiro, Avenida Athos da Silveira Ramos, no 149, Rio de Janeiro, Rio de Janeiro 21941-909, Brasil; ∥ Laboratório de Química Medicinal, Síntese Orgânica e Modelagem Molecular (LaQMedSOMM), Departamento de Química e Bioquímica, Instituto de Química, Universidade Estadual Paulista (UNESP), Rua Prof. Francisco Degni, 55, Araraquara, São Paulo 14800-060, Brasil

## Abstract

Ureases are metalloenzymes found in plants, algae, fungi, and bacteria that are responsible for hydrolyzing urea into carbamate and ammonia. The bacterium Helicobacter pylori, which is associated with gastrointestinal disorders, produces large amounts of urease to neutralize stomach acidity. The rising antibiotic resistance of H. pylori presents a significant challenge for eradication efforts, highlighting the need for novel therapeutic strategies. In this study, we explored the LaSMMed chemical library to identify new urease inhibitors. Virtual screening identified six thioureas derived from cinnamic acid (**LaSMMed 37–46**), demonstrating urease inhibition rates ranging from 13% to 82%. The most potent compound, **LaSMMed 42** (%*I* = 82%), was selected as a lead structure for designing a new series of arylthioureas (**LaSMMed 122–126)**. These derivatives exhibited impressive inhibitory activity, with 84% and 88% inhibition rates. Their IC_50_ values ranged from 0.464 to 0.575 mM, and their inhibition constants (ki) were between 0.080 and 0.130 mM, indicating competitive inhibition for **LaSMMed 125** and mixed-type inhibition for **LaSMMed 122–124** and **LaSMMed 126**. Molecular modeling studies provided insights into the structure–activity relationships and potential binding interactions, supporting their role as promising candidates for the development of new urease-targeting agents.

## Graphical abstract

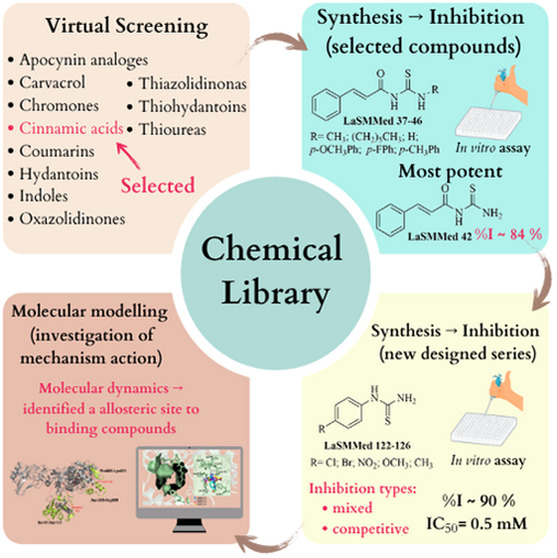

## 1 Introduction

Ureases are a type of metalloenzyme that belongs to the enzyme families amidohydrolase and phosphodiesterase. Their exclusive use of Ni^2+^ ions within their active sites sets them apart from other metal-dependent hydrolases. Ureases can be found in various organisms such as plants, algae, fungi, and bacteria. However, they are not present in animals.^1^ These enzymes hydrolyze urea to form carbamate and ammonia.^2^

Ureases play critical roles in pathogen survival and virulence. Interestingly, ureases found in various microorganisms and plants exhibit significant similarity, despite their polypeptide chain composition differences. The catalytic amino acid residues of vegetal urease from Canavalia ensiformis (CEU), H407, H409, K490, H492, H519, H545, and D633, were found to be identical to their respective equivalents in bacterial urease Helicobacter pylori (HPU).^3^

H. pylori is a bacterium that can colonize the human stomach and cause various gastrointestinal problems. It adapts to the harsh stomach environment by producing a significant amount of urease that neutralizes pH levels.^4^ H. pylori infection is a significant global health concern, particularly in low-income regions, and is associated with an increased risk of gastric cancer.^5–7^ Eradicating H. pylori is challenging because of antibiotic resistance, which requires the development of innovative treatment strategies. Our research group has developed several planned compounds using medicinal chemistry strategies. The LaSMMed chemical library contains over 300 diverse compounds, including coumarins, indoles, chromones, carvacrol derivatives, cinnamic acid derivatives, oxazolidinones, thiazolidinones, apocynin derivatives, hydantoins, thiohydantoins, and thioureas (Figure 1). These compounds are potential pharmacophoric groups for various biological activities, including antibacterial,^8,9^ anticancer,^10^ antiparasitic,^11–14^ antifungal,^15^ antioxidant,^16^ or anticholinesterase inhibition.^17^ In addition, we recently reported compounds such as coumarins,^18^ thiohydantoins, and hydantoins^19^ as potent inhibitors against C. ensiformis urease. We chose the LaSMMed chemical library for virtual screening because its compounds exhibit notable biological activity with at least one subunit linked to antimicrobial effects. We aim to identify potential hits that inhibit bacterial ureases in pathogenic microorganisms like H. pylori. In this study, we identified potential hits from the LaSMMed chemical library and then synthesized and assessed the *in vitro* antiureolytic activity. Additionally, we designed and synthesized a new series of five arylthioureas with characteristics that enhance urease inhibition. The new series underwent evaluation *in vitro* for its inhibitory potential and mechanism of action. Finally, we conducted molecular modeling studies to gain insights into the structural activity relationship of this compound class (Figure 1).

**Figure 1:**
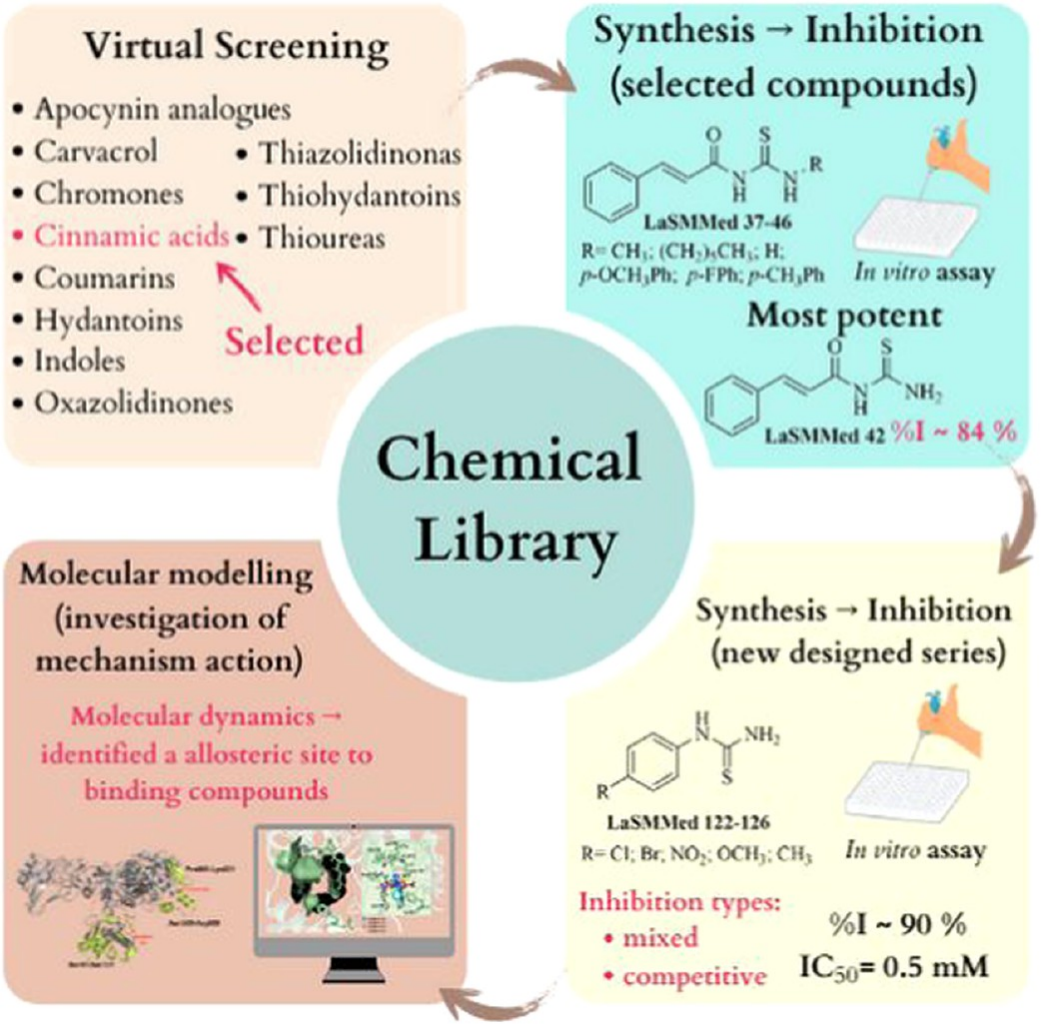
Structural design, experimental, and *in silico* approaches were applied to evaluate **LaSMMed 37–46** and **LaSMMed 122–126** derivatives as potential urease inhibitors.

## 2 Results and Discussion

### 2.1 Virtual Screening on H. pylori Urease

The 3D structure of H. pylori urease (**HPU**, PDB ID: 6ZJA) used in our studies has a cocrystallized inhibitor of the hydroxycarbamide class (**DJM)**, with a molecular volume of 244.12 Å^3^. We made the virtual screening of 130 substances (Table S1) available in our in-house chemical library, which has a molecular volume ranging from 90.25 to 280.06 Å^3^. The selection criteria for our substances were based on their interactions with key residues K219, H221, H248, A278, G279, M317, C321, H322, M366, and I467­(R), involved in interactions with **DJM** and with the catalytic Ni^2+^ ions (Ni601 and Ni602) (Tables S2–S15).

Our findings revealed that six substances from the LaSMMed chemical library interacted with 9 or 10 of the above-mentioned key residues from **HPU**. These substances are derivatives of *trans*-cinnamic acid, differing structurally due to the aliphatic groups (**LaSMMed 37**, **40**, and **42**) or the −4-substituted benzene rings (**LaSMMed 44**, **45**, and **46**) containing electron-donating (methoxy or methyl) or withdrawing groups (fluorine). All of them are characterized by a common thiourea subunit in their structures (Figure 2).

**Figure 2:**
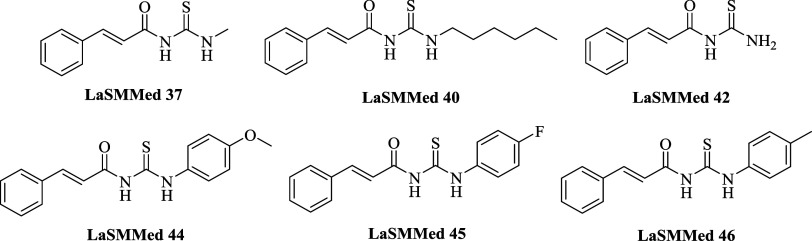
Substances from the LaSMMed chemical library were selected in the virtual screening process as possible H. pylori urease inhibitors.

In general, all selected substances interacted with both Ni^2+^ ions and formed crucial hydrogen interactions with K221, a modified lysine pivotal for enzymatic activity,^20^ as well as H221 and H248 histidines, which are located close to the center of the active site. Additionally, all compounds interacted with residues H136, H138, A169, H247, L318, and D362.

The *trans*-cinnamic acid derivatives **LaSMMed 37**, **40**, and **42** with aliphatic substituents made conventional hydrogen bonds with A169, K219, H221, and G279. Electrostatic interactions of the sulfur in the thiourea subunit with the imidazole ring from H136, H138, H221, and H248, along with metal–acceptor interactions between nickel ions and sulfur, were also present for all substances (Figure 3A–C). Minor interaction differences were observed for **LaSMMed 37**, **40**, and **42**. **LaSMMed 203** approached H138, enabling a hydrogen bond interaction (S-HN) at 3.4 Å (Figure 3A). **LaSMMed 37** distanced itself from D362, losing the hydrogen bond interaction but generating a hydrophobic interaction type π-alkyl between H322 and the hexyl CH (Figure 3A). **LaSMMed 42** closely resembled **LaSMMed 37**, with the most significant difference being the loss of the hydrogen bond interaction with H138 (Figure 3C).

**Figure 3:**
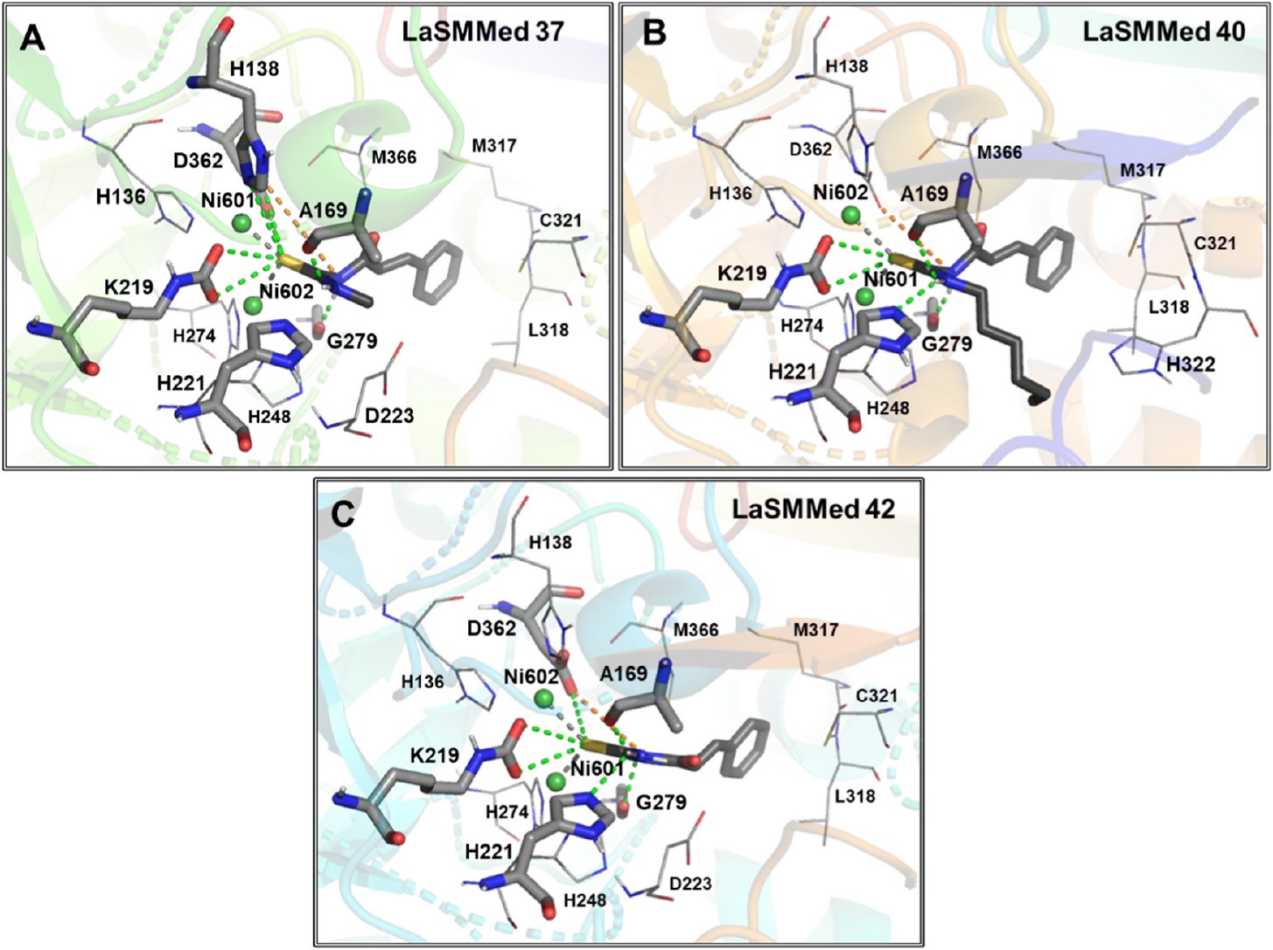
Interactions of the substances (A) **LaSMMed 37**, (B) **40**, and (C) **42** with HPU. The gray sticks represent residues involved in hydrogen bonding, and the lines represent hydrophobic ones. Hydrogen bonds or π-S are shown by dashed green lines and π-alkyl in orange lines.

Thioureas featuring aromatic substituents **LaSMMed 44**, **45**, and **46** exhibited interactions similar to those highlighted for aliphatic ones. However, the phenyl subunit in the substances contributed more to ligand–protein complementarity, allowing electrostatic interactions with residues D223 (π-anion) and A169 (π-alkyl) (Figure 4A–C).

**Figure 4:**
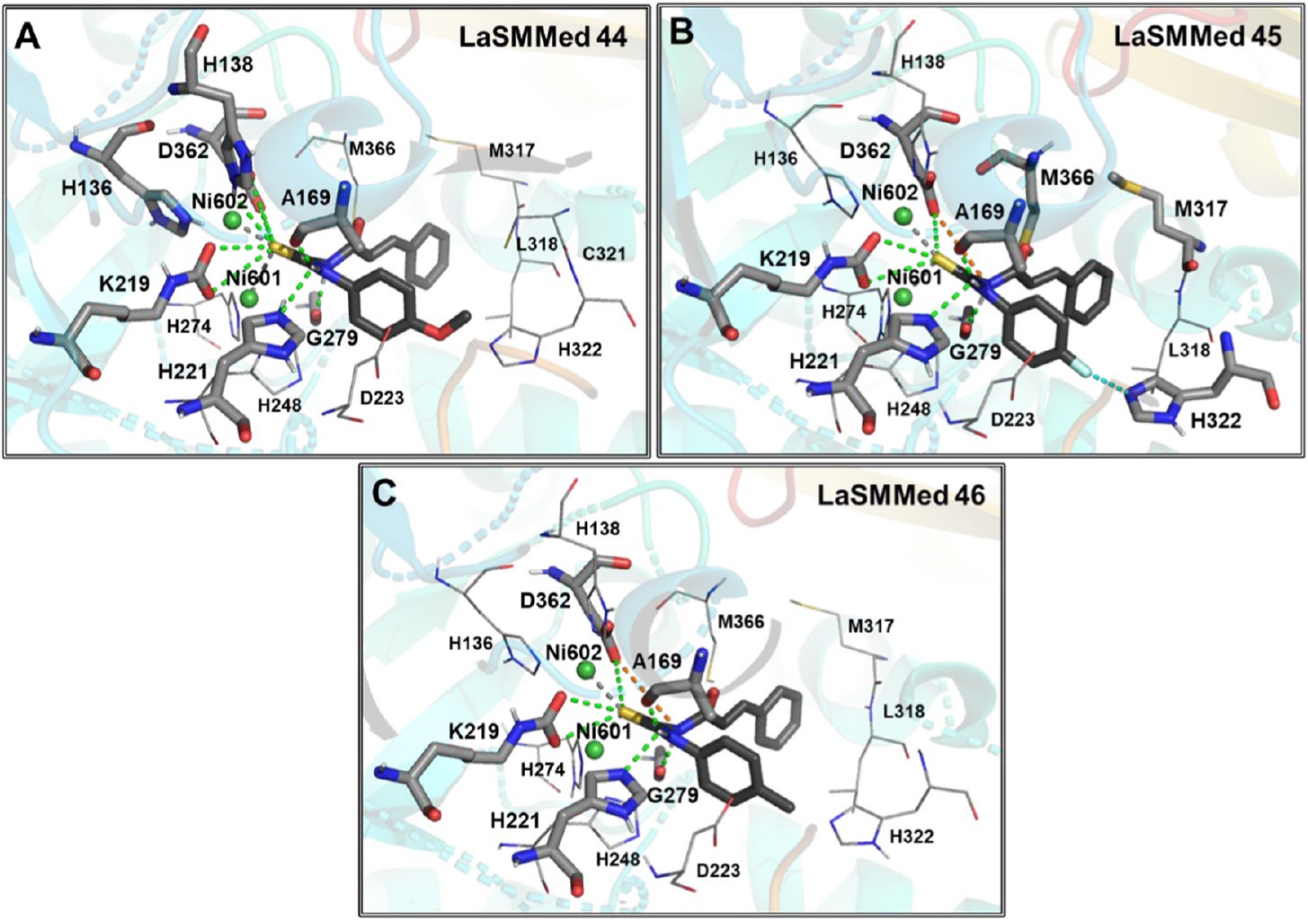
Interactions of the substances (A) **LaSMMed 44**, (B) **45**, and (C) **46** with HPU. The gray sticks represent residues involved in hydrogen bonding, and the lines represent hydrophobic ones. Hydrogen bonds or π-S are shown by dashed green lines, halogen interaction is shown in cyan, and π-alkyl is shown in orange lines.

**LaSMMed 44** established a hydrogen bond (S-HN) with H138 at a distance of 2.9 Å (Figure 4A). **LaSMMed 45** featured a π-S interaction between the benzene ring of the *trans*-cinnamic acid subunit and the sulfur of M317, along with a π-alkyl interaction with the carbon chain of M366 (Figure 4B). **LaSMMed 46** adopted a pose allowing for a π-S interaction between the benzene ring and the sulfur of M366, along with a π-alkyl interaction with M317, L318, and C321 (Figure 4C). It was also observed for **LaSMMed 44**. The inclusion of a fluorine atom in **LaSMMed 45** facilitated a halogen interaction with the nitrogen of H322, which served as a Lewis base. In contrast, the other derivatives exhibited a π-alkyl interaction between the imidazole of H322 and the methyl present in the ligands (**LaSMMed 44** and **46**).

### 2.2 Synthesis of **LaSMMed 37–46** Derivatives Identified by Virtual Screening

The synthesis of six thioureas derived from cinnamic acid (**LaSMMed 37–46**) was conducted using a methodology previously described by our research group.^21^ This process involved three steps carried out in a one-pot protocol, where primary amines were reacted with cinnamoyl isothiocyanate. First, the *trans*-cinnamic acid chloride was synthesized (Table 1, step a). Next, ammonium thiocyanate was added to form the corresponding cinnamoyl isothiocyanate (Table 1, step b). Finally, the appropriate primary amine was introduced into the reaction medium, resulting in the desired thioureas (**LaSMMed 37–46**) with yields ranging from 62 to 90% (Table 1, step c).

**Table 1:**
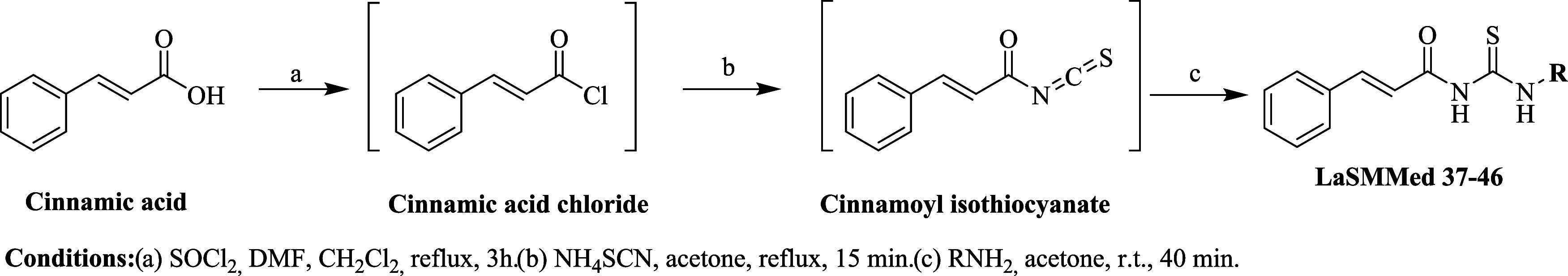
Results of the Synthesis of Thioureas Derived from Cinnamic Acid **LaSMMed 37**, **40**, **42**, **44**, **45**, and **46**

**compound**	***R***	**yield (%)**
**LaSMMed 37**	CH_3_	69
**LaSMMed 40**	Hexyl	62
**LaSMMed 42**	H	74
**LaSMMed 44**	4-OCH_3_Ph	75
**LaSMMed 45**	4-FPh	72
**LaSMMed 46**	4-CH_3_Ph	90

The structures of **LaSMMed 37–46** derivatives were confirmed through ^1^H and ^13^C NMR analyses (Figures S1–S9, Supporting Information). In the ^1^H NMR spectra, characteristic signals indicated the presence of products: an NH singlet appeared between 8.5 and 10 ppm, while the α and β carbonyl hydrogens were observed as doublets at 6.5–7.0 and 7.5–8.0 ppm, respectively. Additionally, the methine hydrogens of benzene rings were detected in the range of 7.0–8.0 ppm. The ^13^C NMR spectra displayed α,β-carbon signals at 120 and 145 ppm, aromatic carbons above 110 ppm, and quaternary carbons between 180 and 170 ppm.

### 2.3 Antiureolytic Activity of the **LaSMMed 37–46** on C. ensiformis Urease

The compounds **LaSMMed 37–46**, which were selected during the virtual screening process, underwent the indophenol reaction to assess their potential to inhibit the enzymatic activity of C. ensiformis urease (**CEU**). This highly purified commercial urease is commonly used for *in vitro* experiments.^22^ Among the substances evaluated, the monosubstituted derivative **LaSMMed 42** showed an impressive **CEU** inhibition of 82%, which was statistically comparable to the thiourea standard that also achieved 84% inhibition (Table 2). The disubstituted cinnamic acid derivatives, **LaSMMed 37** and **40**, both with aliphatic substituents, demonstrated moderate inhibition percentages (%I) ranging from 30% to 40%. Notably, fluorine- and chlorine-substituted cinnamic acid derivatives have been shown to exhibit variable bioactivity depending on their substitution patterns,^23^ which may explain the divergent inhibition rates observed here. In contrast, aromatic derivatives **LaSMMed 45** (4-FPh) and **46** (4-CH_3_Ph) displayed lower inhibition rates of 24 and 13%, respectively (Table 2). Additionally, compound **LaSMMed 44** was insoluble under the tested conditions, preventing it from being analyzed (Table 2).

**Table 2:**
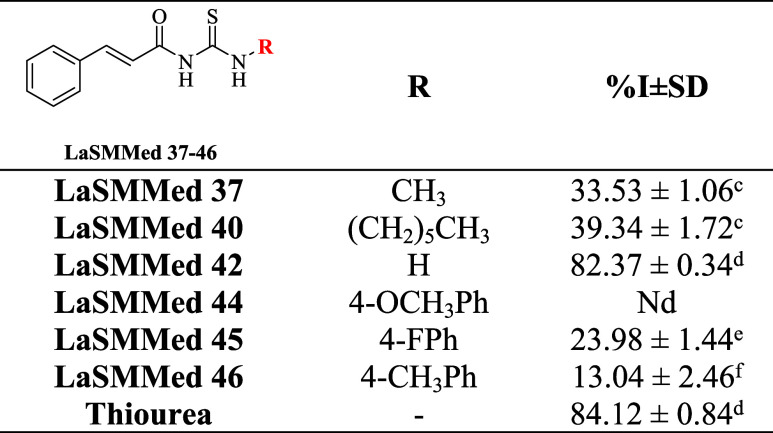
Inhibitory Percentage (%I) of **LaSMMed 37-46** and Standard Thiourea against **CEU^a^**

a SD: standard deviation of experiments performed in triplicate; Nd: not determined (insoluble). Different letters indicate a significant difference between values using the Scott–Knott test (*P* < 0.05).

The results of the structure–activity relationship indicate that the primary thiourea subunit (without substituents) may freely enter the enzyme’s active site, interacting with Ni^2+^ ions and key amino acid residues. This interaction inhibits urea catalysis and ammonia release. As a result, due to its chemical structure, **LaSMMed 42** shows promise as a prototype substance for designing new inhibitors. Accordingly, we propose designing a new small series of five arylthioureas that can serve as urease inhibitors, and we synthesized them to validate this hypothesis.

### 2.4 Synthesis and Characterization of Arylthioureas **LaSMMed 122–126**

We synthesized a concise set of monosubstituted arylthioureas (**LaSMMed 122–126**) by incorporating groups with varying stereoelectronic characteristics at the 4*-*position of the benzene ring. Initially, the respective aniline reacted with concentrated hydrochloric acid (Table 3, step a). Following this, a solution of ammonium thiocyanate was added to the reaction mixture, forming arylthiourea derivatives **LaSMMed 122–126** with yields ranging from 38 to 48% (Table 3, step b).

**Table 3:**
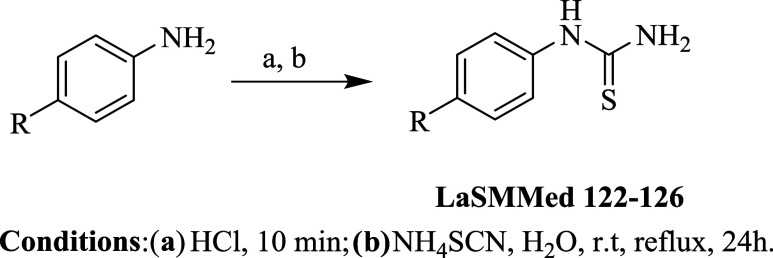
Results of the Synthesis of Arylthioureas **LaSMMed 122-126**

**compound**	***R***	**yield (%)**
**LaSSMed 122**	Cl	35
**LaSSMed 123**	Br	50
**LaSSMed 124**	NO_2_	38
**LaSSMed 125**	OCH_3_	48
**LaSSMed 126**	CH_3_	46

The substances were characterized by using ^1^H and ^13^C NMR spectroscopy (Figure S10–S13). Characteristic signals include a singlet at 9.5–10.3 ppm indicating disubstituted NH, as well as four aromatic hydrogens between 7.0 and 7.5 ppm. Additionally, a singlet at 3.5 ppm was observed, which is characteristic of primary amine hydrogens. In the ^13^C NMR spectra, signals were noted at 180 ppm, which are typical for carbonyls and thiones, along with aromatic methine CH signals ranging from 110 to 140 ppm.

### 2.5 Antiureolytic Activity of the **LaSMMed 122–126** on C. ensiformis Urease

The results from the enzyme inhibition tests on the **CEU** assay indicate that arylthiourea derivatives exhibit excellent urease inhibitory potency, with inhibition percentages nearing 90% (Table 4). The derivatives **LaSMMed 122–126** and **LaSMMed 126** showed no statistically significant differences, suggesting that the substitutions on the benzene ring did not affect the antiureolytic activity within this series (Table 4). However, the derivative **LaSMMed 125**, which contains a powerful electron-donating group (OCH_3_), demonstrated a notable difference in its inhibition value of 84% compared to the other derivatives, but it was statistically equal to that of the standard thiourea (Table 4). Notably, nitrogen-containing heterocycles (e.g., flavonoid analogues) have shown that electronic and steric modifications to the B-ring can significantly influence enzyme binding affinity, which may explain the enhanced activity of **LaSMMed 124** (NO_2_-substituted) compared to other derivatives.^24^

**Table 4:**
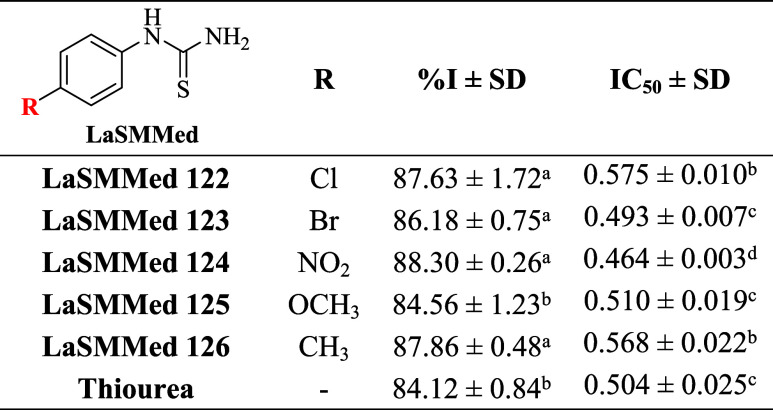
Inhibitory Activity (%I) and IC_50_ (mM) of **LaSMMed 122-126** and Standard Thiourea against **CEU^a^**

a IC_50_: half-maximum inhibitory concentration. SD: standard deviation of experiments performed in triplicate. Different letters indicate a significant difference between values using the Scott–Knott test (*P* < 0.05).

We also assessed the potency of derivatives by determining their half-maximum inhibitory concentration (IC_50_). Among the arylthioureas, the nitro-substituted derivative **LaSMMed 124** exhibited the highest enzymatic inhibition, with an IC_50_ of 0.464 mM, outperforming the standard thiourea (IC_50_= 0.504 mM). **LaSMMed 123** and **125** exhibited IC_50_ values that were statistically comparable to those of thiourea (Table 4). In contrast, the arylthioureas with chloro and methyl substituents, **LaSMMed 122** and **126**, demonstrated the lowest inhibitory potency for **CEU**, with IC_50_ values nearing 0.575 mM (Table 4).

We investigated the mechanism of action of **LaSMMed 122–126** derivatives using Lineweaver–Burk plots to determine the inhibition constant values (*K_i_*), which are calculated from the slopes of each line graph. The results of Lineweaver–Burk plots indicate that, except for **LaSMMed 125**, which acts as a competitive inhibitor (Figure 4D), all other substances demonstrate mixed inhibition against **CEU.** This is evident from the graphs, where there is interception at different points on the *Y*-axis in the second quadrant of the *X*-axis^25^ (Figure 5A–C and E).

**Figure 5:**
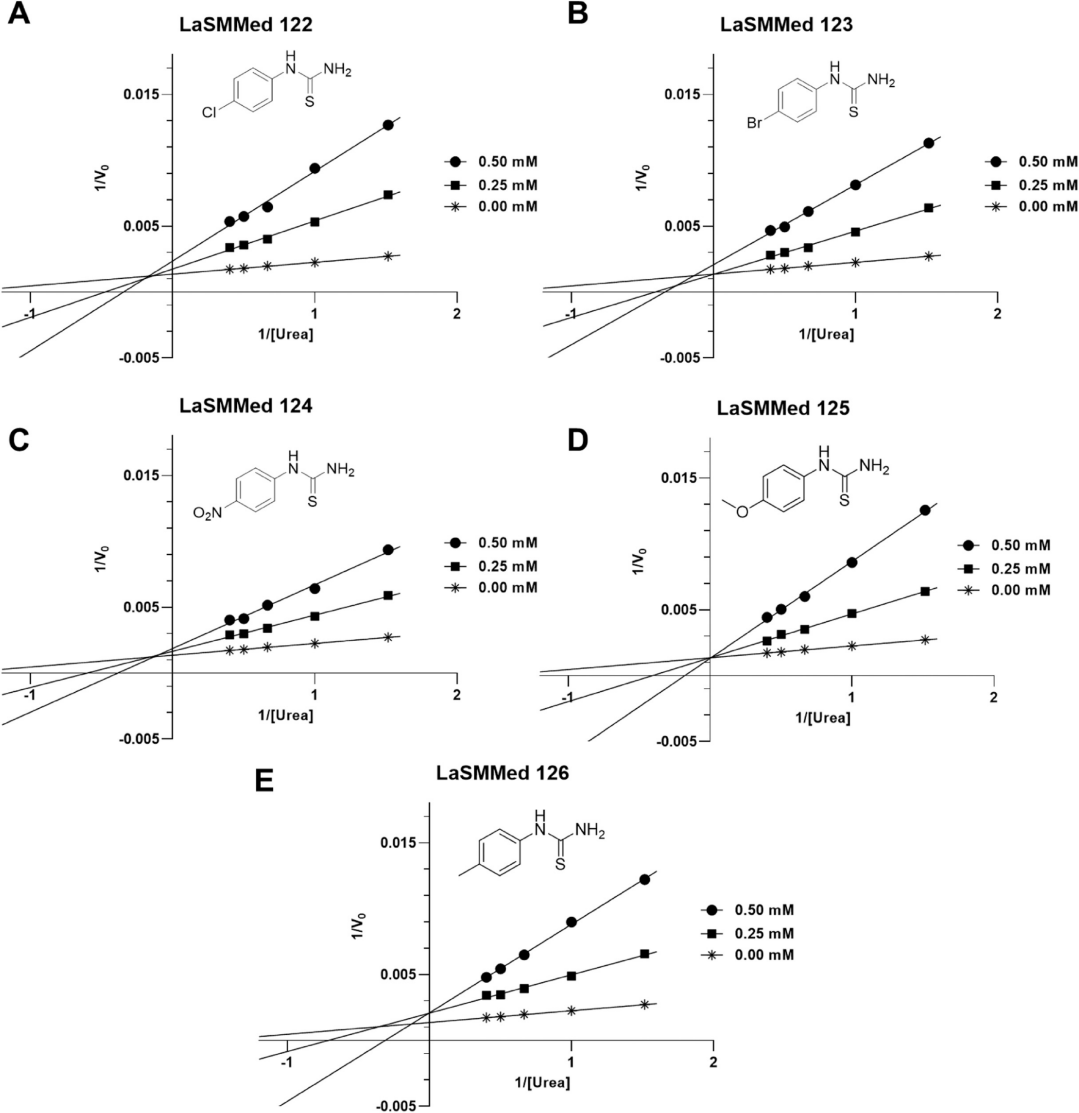
Urease inhibition mode from Lineweaver–Burk plots of the reciprocal reaction rate vs the reciprocal of the substrate (urea) in the absence (*) and in the presence of 0.50 mM (■) and 0.25 mM (•) of compounds **LaSMMed 122** (A), **LaSMMed 123** (B), **LaSMMed 124** (C), **LaSMMed 125** (D), and **LaSMMed 126** (E).

The derivatives demonstrated *V*_max_ values ranging from 732 to 741 μmol of NH^4+^ per minute per milligram of protein, while *K*_m_ values ranged from 0.65 to 0.67 mM (Table 5). Among the evaluated substances, **LaSMMed 125** demonstrated the highest affinity for urease with a *K*_*i*_ of 0.08 mM, closely followed by **LaSMMed 122** that had a *K*_*i*_ of 0.09 mM. In contrast, **LaSMMed 123**, **124**, and **126** all showed similar *K*_*i*_ values of 0.100 mM (Table 5).

**Table 5:**
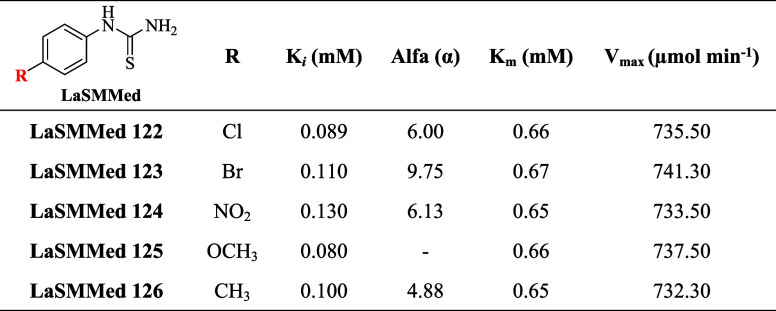
Effect of Arylthioureas **LaSMMed 122-126** on **CEU** Kinetic Parameters

In addition, the α parameter was calculated for all mixed inhibitors (**LaSMMed 122–124** and **LaSMMed 126**), and the results showed values significantly greater than 1. This indicates that these inhibitors have a stronger preference for binding to the free enzyme rather than to the enzyme–substrate complex. Consequently, the mixed model tends to resemble competitive inhibition.^26^

### 2.6 Molecular Docking and Molecular Dynamics of Arylthioureas **LaSMMed 122–126** in **CEU**

Based on our findings, most inhibitors preferentially form an enzyme–inhibitor complex through a mixed-type mechanism. This process blocks the formation of the urease–urea complex, thereby reducing the enzymatic activity. To further investigate this, we conducted an *in silico* analysis to explore the interaction between **CEU** and **LaSMMed 122–124** and **126** at the allosteric site. Our goal was to identify the potential interaction characteristics of this class of compounds with the molecular target.

In our previous research, we mapped the structure of **CEU**, and we identified an allosteric site located in the region referred to as the “hammer handle”, which is at the junction between the αβ and β domains.^25^ The molecular docking results of inhibitors **LaSMMed 122–124** and **126** indicated that they exhibited similar binding poses within this allosteric site of **CEU** (see Figure 6A).

**Figure 6:**
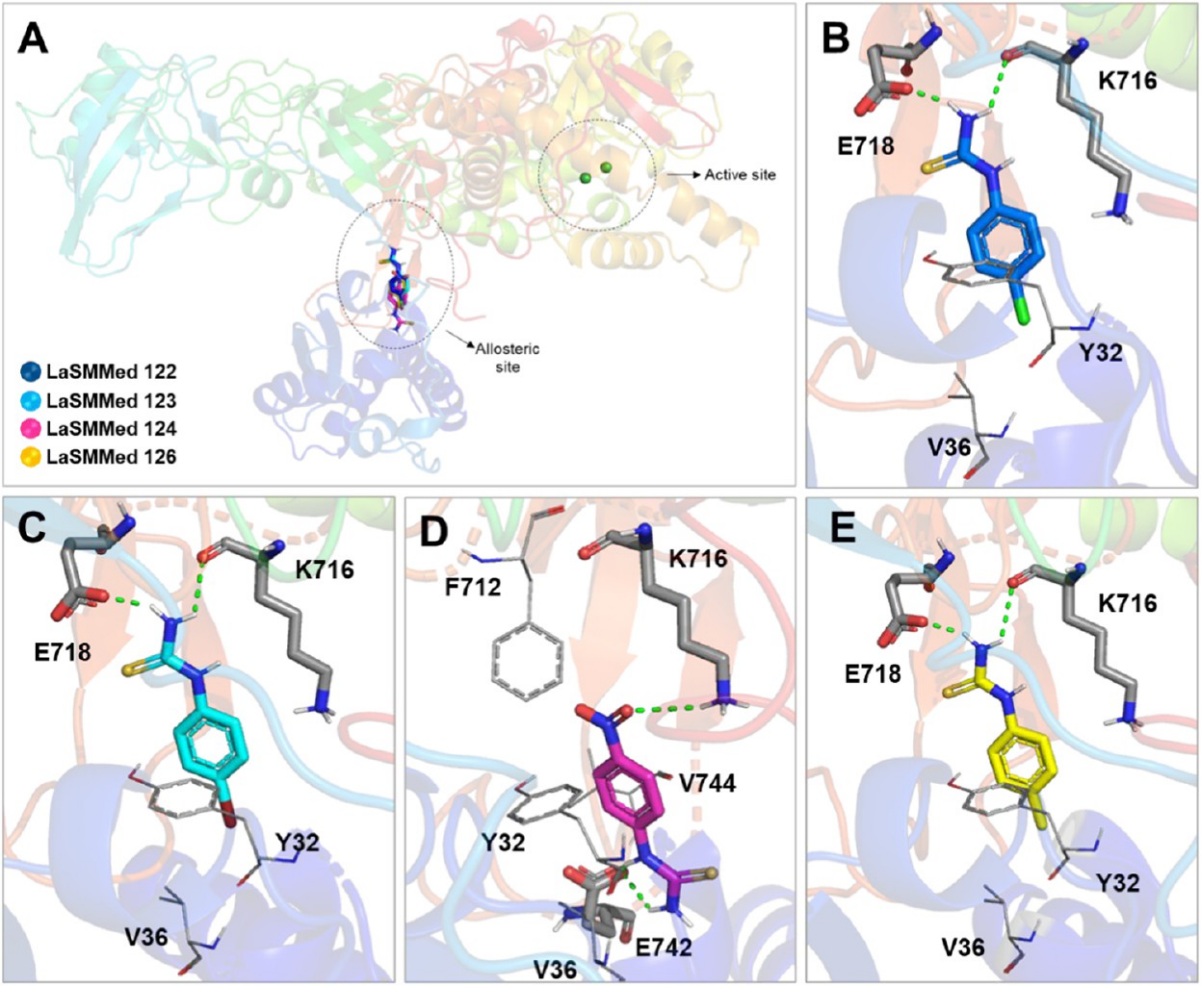
(A) Overlap of inhibitor on the allosteric site from **CEU**. Interactions of substances (B) **LaSMMed 122**, (C) **123**, (D) **124**, and **126** (E) with **CEU**. The gray sticks represent residues involved in hydrogen bonding, and the lines represent hydrophobic ones. Dashed green lines show hydrogen bonds.

With the exception of **LaSMMed 124** (Figure 6D), all other compounds exhibited hydrogen bonding involving the thiourea subunit with E718 and K716. Specifically, for **LaSMMed 122**, the NH group interacted with the carboxylate ion of amino acids E718 and K716 at distances of 1.8 and 2.4 Å, respectively (Figure 6B). A similar interaction occurred for **LaSMMed 123** at distances of 2.0 Å with E718 and 2.3 Å with K716 (Figure 6C). In the case of **LaSMMed 126**, the observed hydrogen bond distances were 1.9 and 2.6 Å for the respective amino acids (Figure 6E). Additionally, all three substances demonstrated hydrophobic interactions between the ligands in position 4 of the aromatic ring and the residues Y32 and V36.

**LaSMMed 124** showed a hydrogen bond interaction between the NH groups of the thiourea subunit and the carboxylate ion of E742 at distances of 1.8 and 2.2 Å. Additionally, another interaction was observed between the NO_2_ group of the ligand and the amine of K716 at a distance of 2.8 Å (Figure 6D). Weaker hydrophobic interactions were observed between the ligand ring and the alkyl groups of V36 and V744 (Figure 6D).

Regarding molecular docking results to **LaSMMed 125** on the active site from the **CEU** enzyme, we identified hydrogen bond interactions involving the thiourea group and the catalytic amino acids KCX490, A440, and D633, which are critical for Ni^2+^ coordination,^3^ as well as with G550. Furthermore, hydrophobic interactions were observed with H407, H492, D494, H519, H593, and R606. Notably, the sulfur atom played a key role in the interaction with the Ni^2+^ ion (Figure 7).

**Figure 7:**
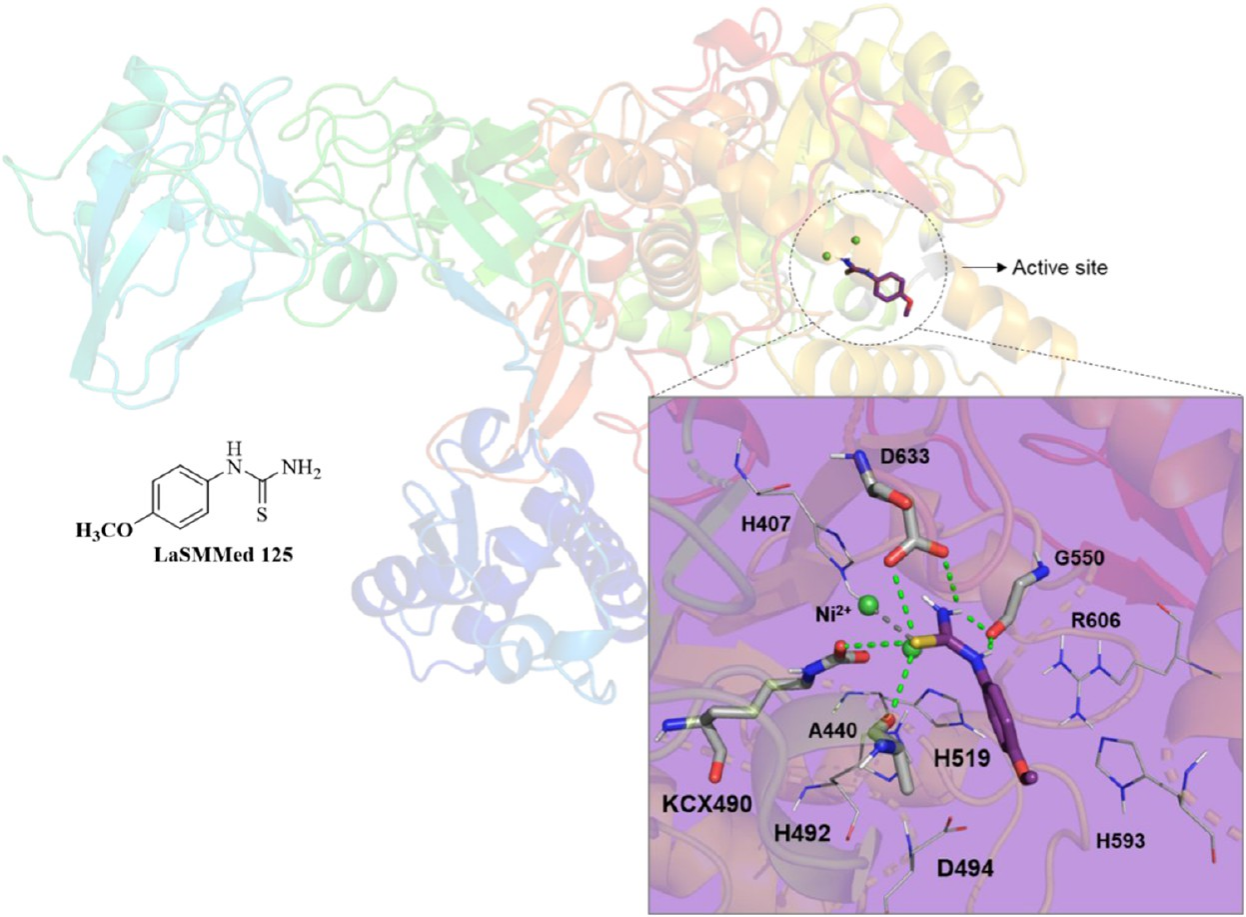
Interactions of the substances **LaSMMed 125** on the active site from **CEU**. The gray sticks represent residues involved in hydrogen bonding, and the lines represent hydrophobic ones. Hydrogen bonds are shown by dashed green lines, and gray dashed lines show metal interaction.

We also included an investigation of the competitive inhibitor **LaSMMed 125** in our analysis. Our findings indicate that the addition of an oxygen atom, resulting in the formation of a methoxy group (*R* = OCH_3_), provides **LaSMMed 125** with the active conformation needed to interact with the active site residues, allowing it to function as a competitive inhibitor. In contrast, its derivative, **LaSMMed 126** (*R* = CH_3_), exhibits mixed-type inhibition.

We investigated the behavior of arylthiourea compounds within an aqueous system at the enzyme’s allosteric site. This study employed molecular dynamics simulations on the enzyme with an unoccupied active site (free site). We analyzed the ligand atoms of **LaSMMed 122–126** using root-mean-square deviation (RMSD) metrics and found that **LaSMMed 123** and **LaSMMed 126** were the most stable compounds at the allosteric site. **LaSMMed 123** had an RMSD value of 2.03 ± 0.75 Å (Figure 8B), while **LaSMMed 126** showed an RMSD value of 2.20 ± 0.56 Å (Figure 8E). In contrast, **LaSMMed 122** and **LaSMMed 124** had higher RMSD values of 5.58 ± 1.16 and 8.34 ± 4.67 Å, respectively. These compounds demonstrated movement in the allosteric site after approximately 50 and 25 ns (Figures 8A,C), compared to their poses, as determined by molecular docking.

**Figure 8:**
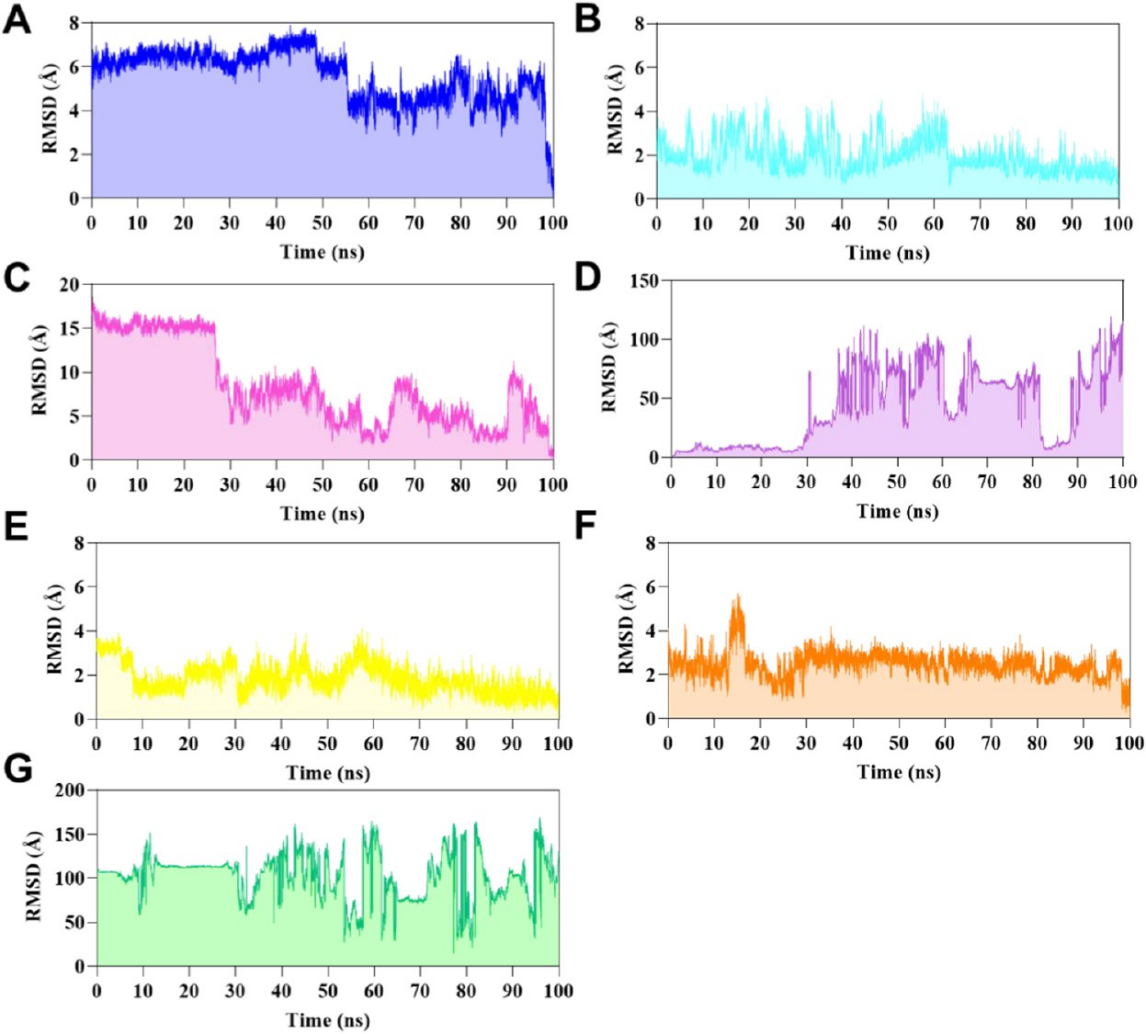
RMSD values during 100 ns of MD simulations based on ligand atoms of (A) **LaSMMed 122**, (B) **123**, (C) **124**, (D) **125**, (E) **126** (free-site), (F) **LaSMMed 126** (occupied-active site with **HAE**), and (G) **HAE** (in the presence of **LaSMMed 126**).

**CEU** has cocrystallized acetohydroxamic acid (**HAE**), a competitive inhibitor structurally similar to the natural urea substrate. This enables us to simulate its behavior in the presence of inhibitors during molecular dynamics studies, thereby verifying the interaction that may indicate binding to the free enzyme rather than the enzyme–substrate complex in cases of mixed model inhibition.

In this way, we analyzed **LaSMMed 126**, which exhibited the lowest α value in *in vitro* tests (α = 4.88). We investigated this ligand along with inhibitor **HAE** to simulate the formation of a potential enzyme–substrate-inhibitor complex. During the simulation, **LaSMMed 126** remained stable at the allosteric site while **HAE** occupied the active site, with an RMSD of 2.46 ± 0.60 Å and only a slight variation observed in 15 ns (Figure 8F). As shown in Figure 8G, **HAE** demonstrated significant variability in its RMSD, with a standard deviation of 102 ± 26 Å. Furthermore, during the simulation, **HAE** disconnected from the complex within the first few nanoseconds (Figure 8G). These findings support the hypothesis that the enzyme–substrate-inhibitor complex does not form in the presence of arylthioureas. Instead, the mixed inhibitors identified in this study preferentially interact with the free enzyme. Finally, we investigated the competitive inhibitor **LaSMMed 125** (Figure 8D) within the enzyme’s active site. It demonstrated stability only in the initial 30 ns of the simulation, followed by a brief period between 82 and 90 ns. During this latter phase, high standard deviation values were observed, with a root-mean-square deviation (RMSD) of 42.7 ± 31.8 Å. During the 100 ns of MD simulations, the RMSD analysis of Cα atoms from the enzyme exhibited stability ranging from 1.75 to 2.42 Å (Figure 9A–E). The ligands in the allosteric site showed lower RMSD values, with **LaSMMed 122** at 1.75 ± 0.23 Å, **LaSMMed 126** (free-site) at 1.86 ± 0.67 Å, **LaSMMed 126** (occupied-site) at 1.92 ± 0.42 Å, and **LaSMMed 123** at 1.92 ± 0.30 Å. In contrast, **LaSMMed 124** had a higher value of 2.42 ± 0.55 Å. The RMSD-Cα atoms to **LaSMMed 125** at the active site were also low, measuring 1.79 ± 0.41 Å (Figure 9F).

**Figure 9:**
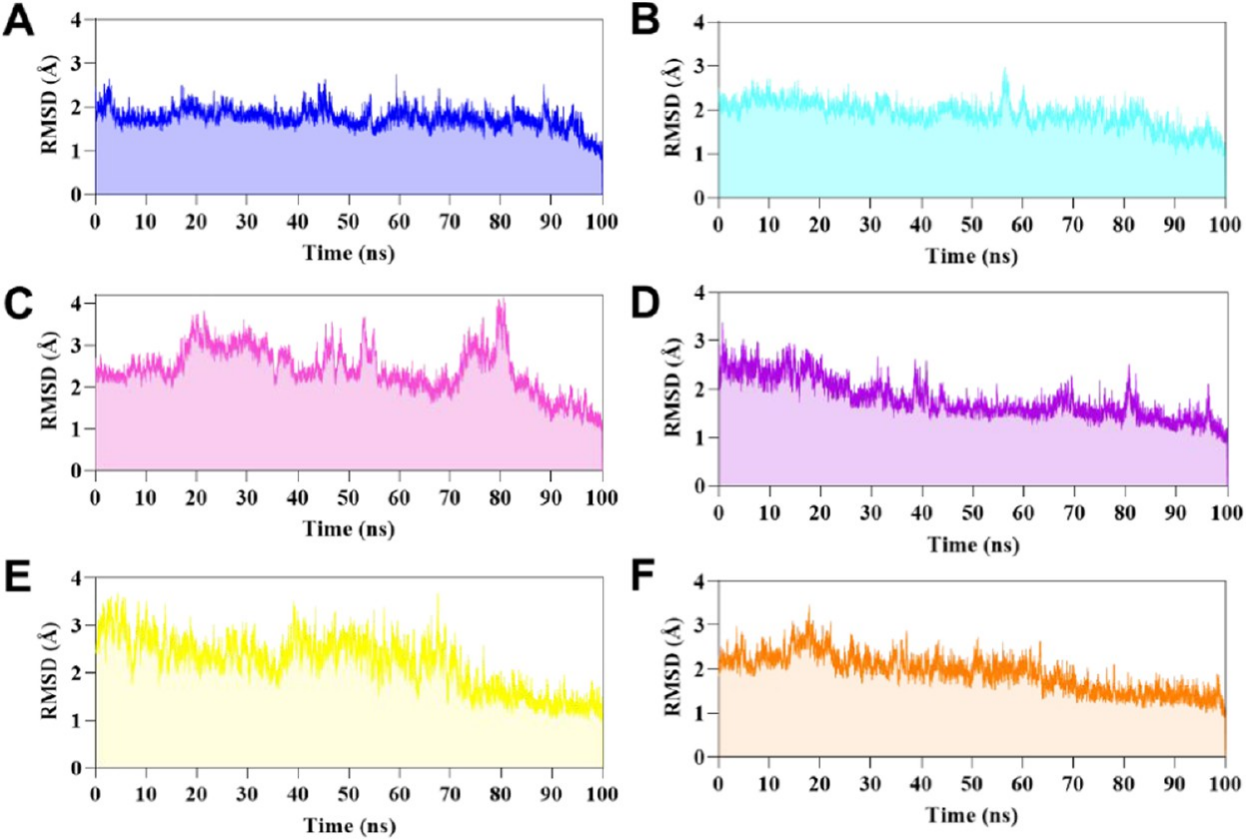
RMSD values during 100 ns of MD simulations based on Cα atoms of protein to **LaSMMed** (A) **122**, (B) **123**, (C) **124**, (D) **125**, (E) **126** (free-site), and (F) **LaSMMed 126** (occupied active site with **HAE**).

Notably, the root-mean-square-fluctuation (RMSF) calculation of all **LaSMMed** inhibitors reveals that specific residues exhibit mobility greater than 3.0 Å for Cα atoms (Figure 10A**–**F). These residues include Ser41 to Asn131 near the allosteric site (Figure 10G) and Asn580 to Arg609, as well as Pro805 to Lys821 within the active site region (Figure 10G).

**Figure 10:**
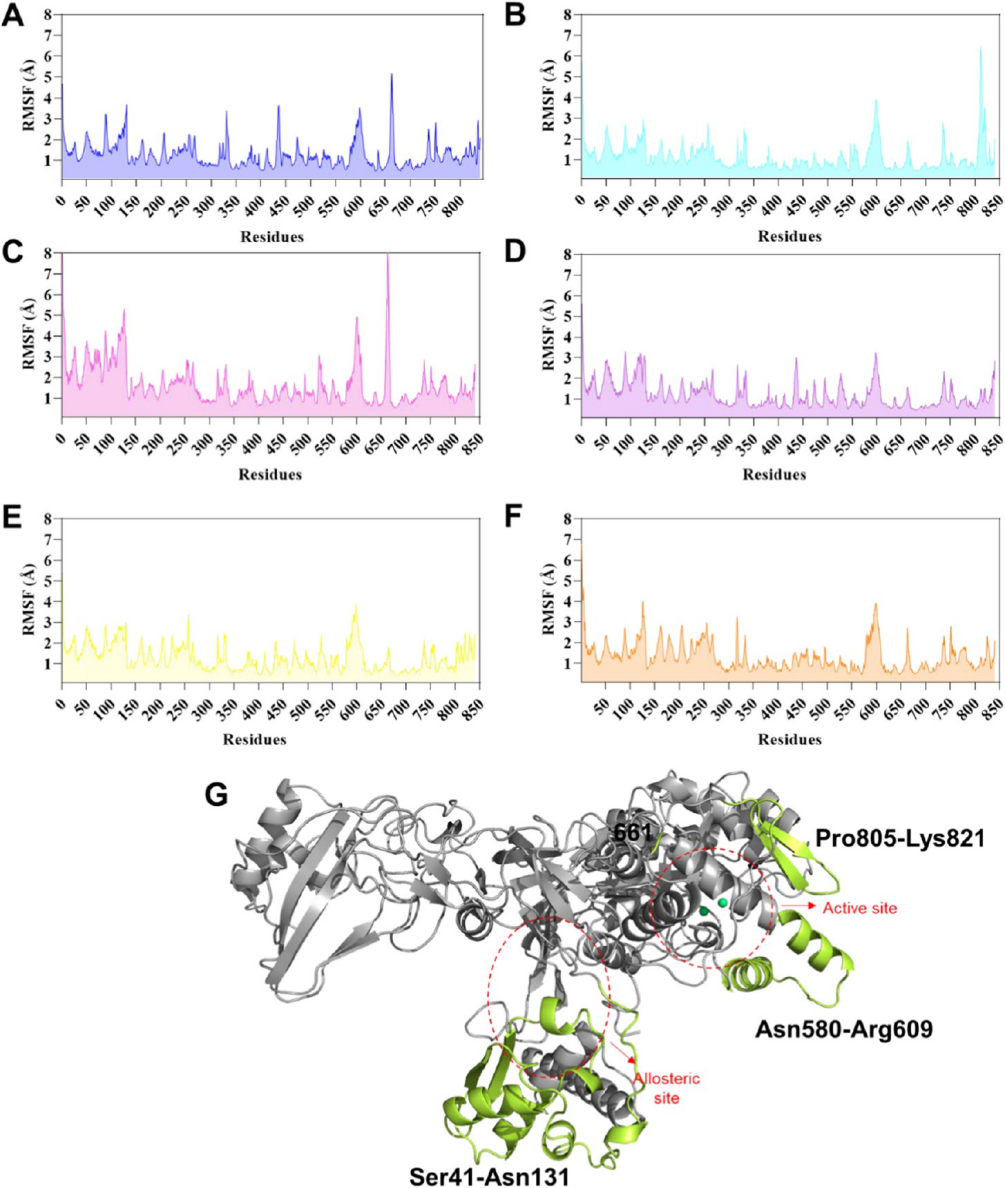
RMSF values during 100 ns of MD simulations based on Cα atoms of protein residues to **LaSMMed** (A) **122**, (B) **123**, (C) **124**, (D) **125**, (E) **126** (free-site), and (F) **LaSMMed 126** (occupied active site with **HAE**). (G) **CEU** 3D structure highlights the residues with the most fluctuations observed in green.

The active site region spanning Asn580 to Arg609 exhibited the most significant fluctuations in the presence of ligands **LaSMMed 122**, **123**, **124**, and **126** (Figure 10A-C,E). Residue 661, located in the C-terminal domain (Figure 10G) where the active site is located,^19^ demonstrated notable RMSF values of 5 and 8 Å in the presence of ligands **LaSMMed 122** and **LaSMMed 124**, respectively (Figure 10A,C). Additionally, ligand **LaSMMed 123** also resulted in RMSF values of approximately 7 Å for amino acids Pro805-Lys821 within the protein loop (Figure 10B,G).

**LaSMMed 126** did not exhibit any significant differences in the RMSF values for residues Asn580–Arg609, whether in the free-enzyme state (Figure 10E) or in the presence of the **HAE** inhibitor (Figure 10F). However, it showed fluctuation values of less than 3 Å for residues Ser41–Asn131, which are located near the allosteric site (Figure 10G). In contrast, the presence of competitive inhibitor **LaSMMed 125** resulted in values up to 3 Å for the regions mentioned above (Figure 10D).

In analyzing hydrogen bonds in the allosteric site, **LaSMMed 122** formed hydrogen bonds with the residues Tyr32, Lys716, and Glu718 within the first few nanoseconds of simulation (Figure 11A). These interactions persisted throughout the simulation, lasting even after 80 ns, despite a change in the ligand′s orientation. The lifetime of the interactions varied between 6 and 85% (Figure 11A).

**Figure 11:**
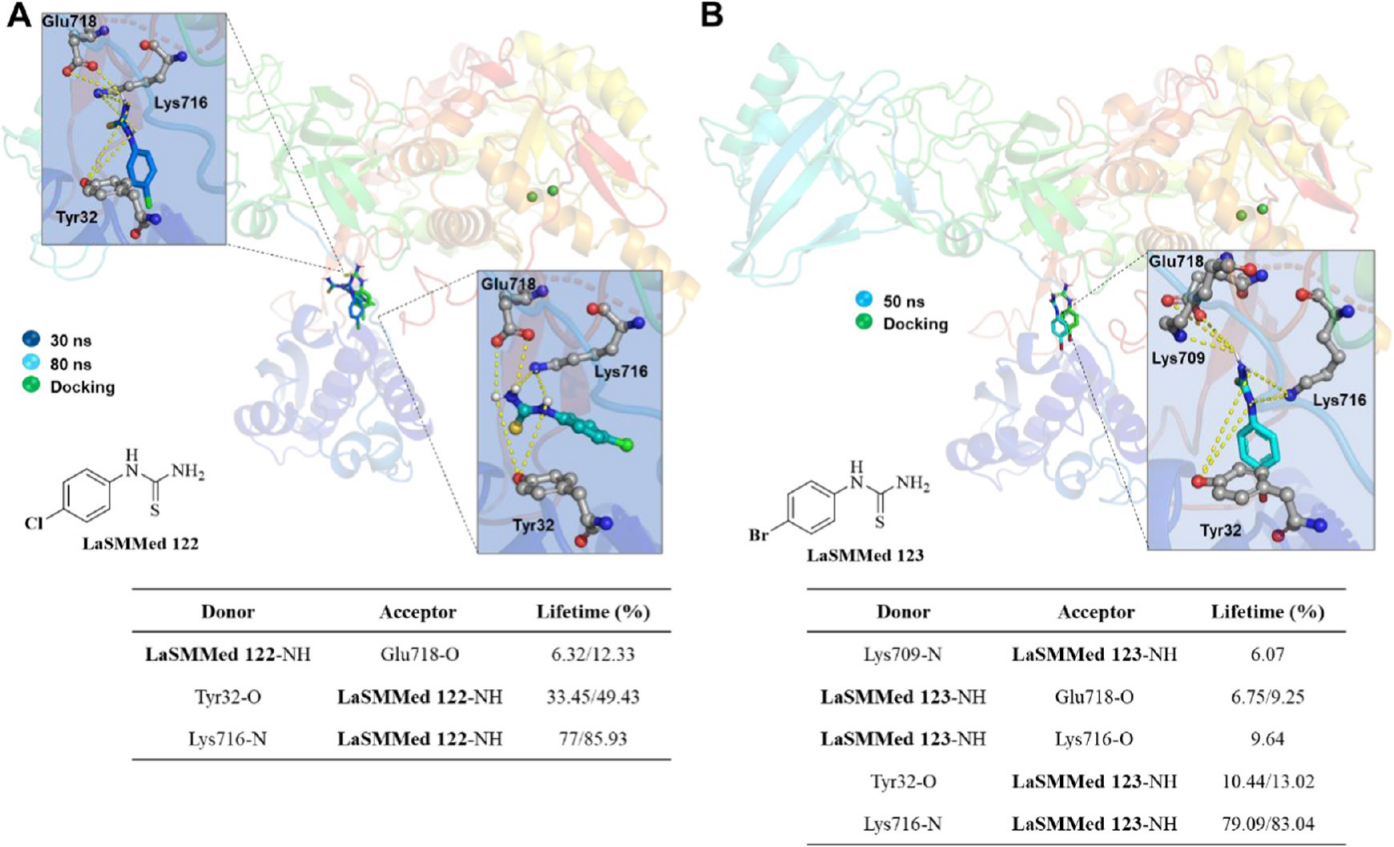
H-bond interactions by molecular dynamics simulations (100 ns) from **LaSMMed-CEU** complexes. (A) **122**; (B) **123.** The table shows donor and acceptor groups involved in the H-bonds and their lifetime (%). The ligands involved in H-bond interactions (dashed yellow lines) are displayed in gray sticks and balls. The pose of ligands by docking results is shown in green sticks for comparison.

**LaSMMed 123** displayed similar interactions to **LaSMMed 122**, with an H-bond lifetime ranging from 6 to 83% (Figure 11B). These interactions persisted throughout the MD simulation. Additionally, **LaSMMed 123** formed another H-bond with Lys-709, likely influenced by the -Br substituent, which is a larger atom (Figure 11B). This interaction positioned the ligand closer to the Tyr32 residue compared with **LaSMMed 122**, where the bonding occurred between residues Lys716 and Tyr32 (Figure 11A). Furthermore, both compounds did not exhibit significant differences in their poses compared to the molecular docking results (in green, Figure 11A,B), as indicated by the RMSD analyses.

In the H-bond analysis of the allosteric site, **LaSMMed 124** initially interacted with Tyr32, Lys709, Asp730, and Lys745 at the start of the simulation. However, there was some movement in its position compared with the docking results (as shown in Figure 12A). After 60 ns, these bonds were lost, and only the interaction with Lys709 remained persistent until the end of the simulation. The lifetimes of these H-bonds varied between 5 and 56.9% (Figure 12A).

**Figure 12:**
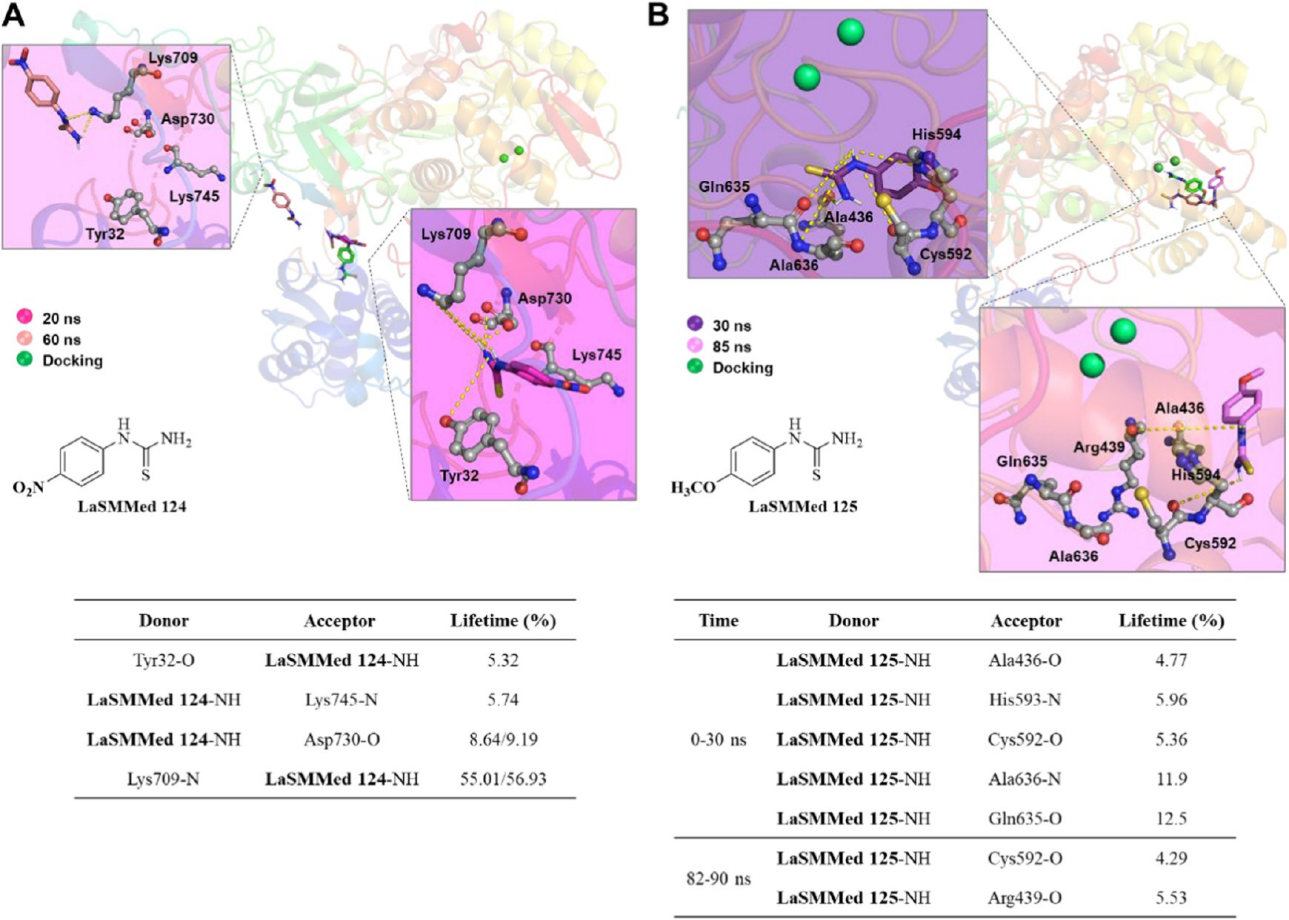
H-bond interactions by molecular dynamics simulations (100 ns) from **LaSMMed-CEU** complexes. (A) **LaSMMed 124** (allosteric site); (B) **LaSMMed 125** (active site). The table shows donor and acceptor groups involved in the H-bonds and their lifetime (%). The ligands involved in H-bond interactions (dashed yellow lines) are displayed in gray sticks and balls. The poses of ligands by docking results are displayed in green sticks for comparison.

Our observations indicated that in the active site, **LaSMMed 125** interacted with residues Ala436, Cys592, His593, Gln635, and Ala636 during the first 30 ns of MD simulation (Figure 12B). However, after this initial interaction, the ligand moved out of the active site and only returned between 82 and 90 ns. During this later period, an H-bond with Arg439, located in the outer cavity of the active site, was established, as noted in the RMSD analysis. Although the H-bond interactions of **LaSMMed 125** had relatively low lifetimes ranging from 5 to 12% (Figure 12B), they were still sufficient to disrupt the catalytic activity of urease, leading to its inhibition.

In the allosteric site, **LaSMMed 126** interacted with Glu718 and Asp730 (21 and 8–74%, respectively) in the enzyme’s free state. This interaction formed strong hydrogen bonds that remained stable throughout the simulation. Additionally, the ligand established two other interactions with shorter lifetimes (ranging from 7 to 17%) with Tyr32 and Lys709. Despite a change in orientation, **LaSMMed 126** remains bound to the same pocket observed during the docking process (Figure 13A).

**Figure 13:**
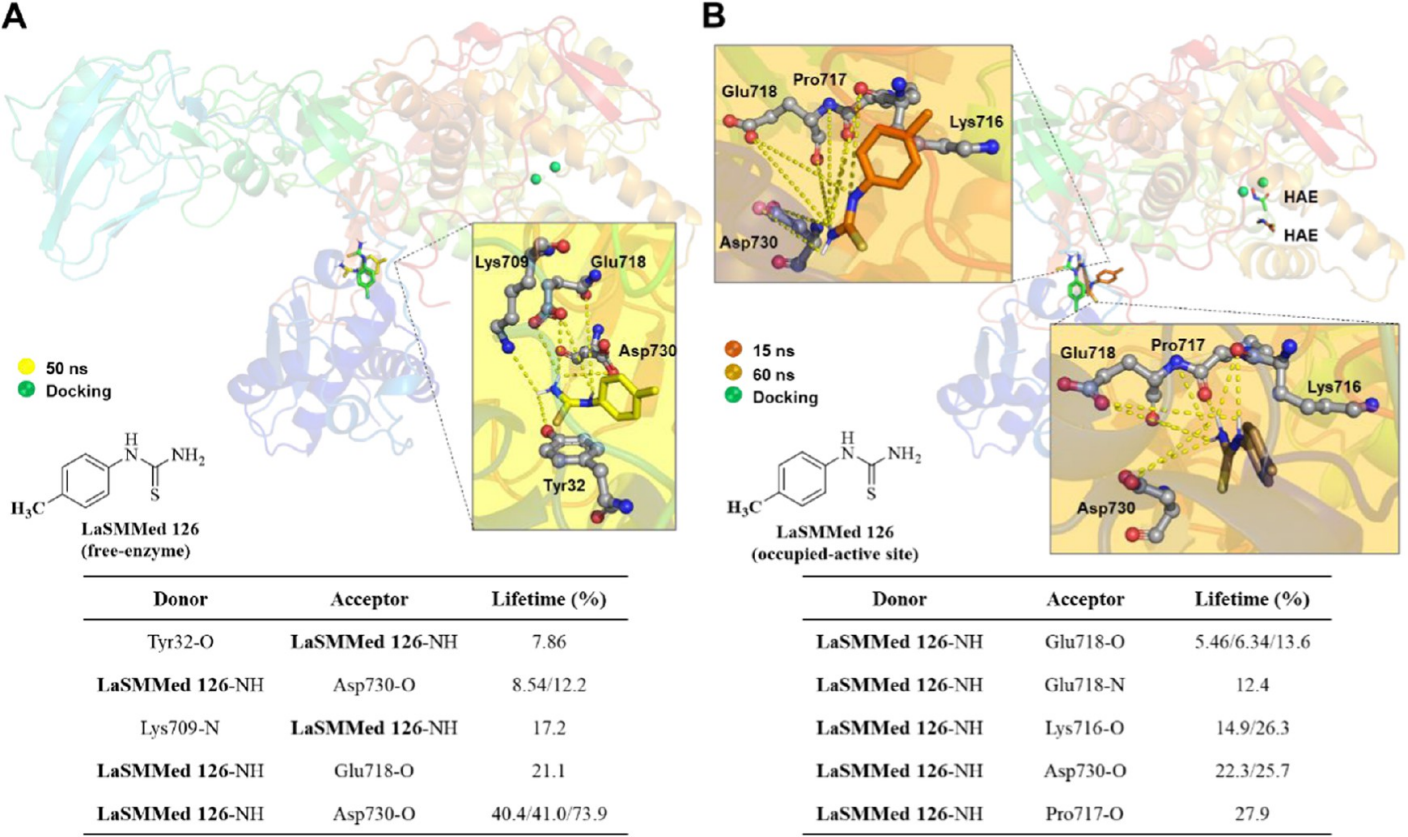
H-bond interactions by molecular dynamics simulations (100 ns) from **LaSMMed-CEU** complexes. (A) **126** (free-site); (B) **125** (occupied-active site). The table shows donor and acceptor groups involved in the H-bonds and their lifetime (%). The ligands involved in H-bond interactions (dashed yellow lines) are displayed in gray sticks and balls. The pose of ligands by docking results is displayed in green sticks for comparison.

In the case of **LaSMMed 126**, when the enzyme’s active site was occupied with **HAE**, interactions with Glu718 and Asp730 were observed, but these interactions had shorter lifetimes, ranging from 5 to 26%. Furthermore, new hydrogen bonds formed with residues Lys716 and Pro717, with lifetimes between 15 and 28% (Figure 13B). It is important to note that the inhibitor **HAE** remained in the active site cavity only during the first 15 ns of the simulation (in orange, Figure 13B) when **LaSMMed 126** was present in the allosteric. Subsequently, **HAE** disconnected from the complex, while **LaSMMed 126** remained connected to the enzyme without any loss of interaction throughout the process (Figure 13b).

A careful examination of the interaction behavior and lifetime percentages of the arylthiourea-type ligands revealed that they tend to interact with urease in its free state. This interaction may prevent the formation of an enzyme–substrate complex, mimicking competitive inhibition. Further research could explore the implications of this mechanism and its potential applications in the development of new urease inhibitors.

We calculated the binding free energy (Δ*G*_bind_) for the complexes throughout 100 ns of simulation using Linear Interaction Energy (LIE).^27^ The average interaction energy trajectory results indicate that the **LaSMMed-CEU** bound state had lower energy than its free state. A similar trend was observed in the electrostatic Coulomb’s interactions, suggesting that binding of **LaSMMed 122–126** to **CEU** was favorable.

The binding energy values were consistent with those of the other molecular dynamics analyses. Among the compounds evaluated at the allosteric site, those with the most extended lifetime hydrogen bond interactions exhibited the highest energy binding values. **LaSMMed 122** had the highest energy value with a Δ*G*_bind_ of −18.3 kJ/mol, followed by **LaSMMed 123** with Δ*G*_bind_ = −17.3 kJ/mol. **LaSMMed 126** had a Δ*G*_bind_ = −14.8 kJ/mol in its free state and a Δ*G*_bind_ = −15.7 kJ/mol in its occupied state, while **LaSMMed 124** showed a Δ*G*_bind_ = −12.1 kJ/mol (Table 6).

**Table 6:**
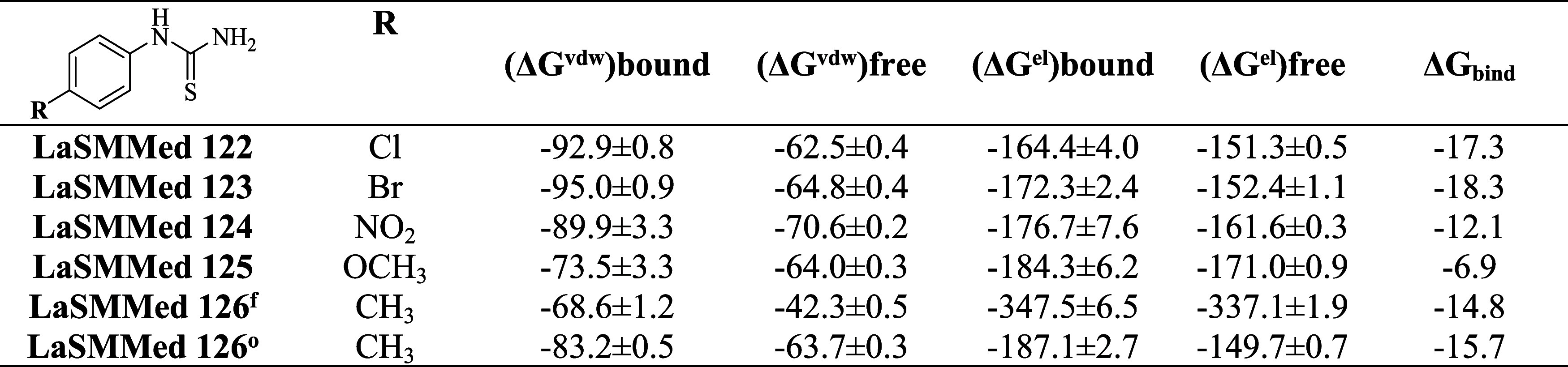
Binding Free Energy (Δ*G*_bind_, Mean ± Standard Deviation in kJ/mol) of **LaSMMed 122-126** Inhibitors on Urease Complexes^a^

a f = free state, o = occupied active site. The vdw refers to nonpolar interactions modeled by a Lennard-Jones potential, and el denotes electrostatic interactions by Coulomb potential; standard coefficients α = 0.161 and β = 0.50.

In the ligand-bonded state, the derivatives evaluated at the allosteric site (**LaSMMed 122**, **123**, **124**, and **126**) showed the most significant Δ*G*_vwd_ values for nonpolar interactions, ranging from −83 to −95 kJ/mol. This can be attributed to the interactions with the Tyr32 residue observed for all of the compounds (Table 6). The most notable contribution to the free energy values came from electrostatic interactions, which were similar across the four derivatives, ranging from Δ*G*_el_ = −164 to −187 kJ/mol. Notably, **LaSMMed 126**, when in the occupied active site state, exhibited an exceptionally high value of Δ*G*_el_ = −337 kJ/mol (Table 6). Compared with the free state, this energetic value may have been influenced by interactions with the Pro717 residue and the lack of interaction with Tyr32. Additionally, the ligand was closer to the residues of Glu718 and Asp730 than was observed in other cases.

**LaSMMed 125** exhibited a low lifetime percentile, resulting in a decreased binding free energy value of Δ*G*_bind_ = −6.9 kJ/mol. The active site of urease primarily consists of acidic or basic residues, including aspartate, histidine, and arginine.^3^ Consequently, the most significant contribution to the free energy value for **LaSMMed 125** arose from electrostatic interactions, with Δ*G* ^el^ = −184.3 kJ/mol, compared to nonpolar interactions, which contributed Δ*G*_vwd_ = −73.5 kJ/mol (Table 6).

Additionally, an analysis was performed to evaluate the energetic contributions of residues involved in hydrogen bonds with inhibitors **LaSMMed 122–124** and **126** at the allosteric site during molecular dynamics simulations. The residues that most significantly contributed to Δ*G*_bind_ were Tyr32, Lys716, Glu718, and Asp730 in most cases. Notably, Tyr32 consistently affected the Δ*G*_vwd_ values across all evaluated complexes. The most substantial energetic contribution came from the Asp730 residue, with values ranging from −7.36 to −151 kJ/mol, followed by Lys716, which ranged from −14.2 to −80.9 kJ/mol (Table 7).

**Table 7:** Binding Free Energy of Amino Acids in Interaction with **LaSMMed 122-126** Inhibitors on Urease Complexes^a^

	**Δ*G***_**bind**_ (mean ± standard deviation in kJ/mol)				
**residues**	**LaSMMed 122**	**LaSMMed 123**	**LaSMMed 124**	**LaSMMed 126** ^b^	**LaSMMed 126** ^c^
**Tyr32**	**-8.77**	**-18.0**	**-9.03**	**-32.8**	**-7.08**
**Lys709**	–4.20	–0.18	–56.9	–0.15	–0.27
**Lys716**	**-51.9**	**-80.9**	**-2.95**	**-21.6**	**-14.2**
**Pro717**	–1.51	–1.40	–0.24	–3.07	–8.56
**Glu718**	**-9.30**	**-8.33**	**-3.22**	**-24.8**	**-9.28**
**Asp730**	**-25.5**	**-20.4**	**-7.36**	**-151**	**-12.5**
**Lys745**	–1.48	–1.89	–0.96	–2.89	–2.85

a In bold, the higher values of **Δ*G*_bind_** to residues by complex.

b Free state.

c Occupied active site.

The analysis of contributions from specific residues supports the finding that in the occupied-site state, **LaSMMed 126** shows a greater energetic contribution from residue Pro717 (−8.56) compared to the free state (−3.07 kJ/mol). Conversely, the contribution from Tyr32 decreases significantly from −32.8 kJ/mol in the free state to −7.08 kJ/mol in the occupied state (Table 7). These changes influence the notably high Δ*G*_el_= −337 kJ/mol observed for the complex. Furthermore, the contribution of Lys709 to the **LaSMMed 124** complex was significantly higher (Δ*G*_bind_= −56.9 kJ/mol) than those of the other complexes (Table 7). This finding is further supported by H-bond analysis, which revealed that the interaction with this residue has a longer lifetime of 56.9% in this complex.

The examination of the seven residues in the allosteric site yielded promising findings. The cavity formed by these residues (Figure 14) exhibits hydrophobic characteristics due to the phenyl ring of Tyr32, the pyrrolidine of Pro717, and the carbonic side chain of the lysine residues. This creates an optimal environment for interaction with the phenyl ring from arylthioureas.

**Figure 14:**
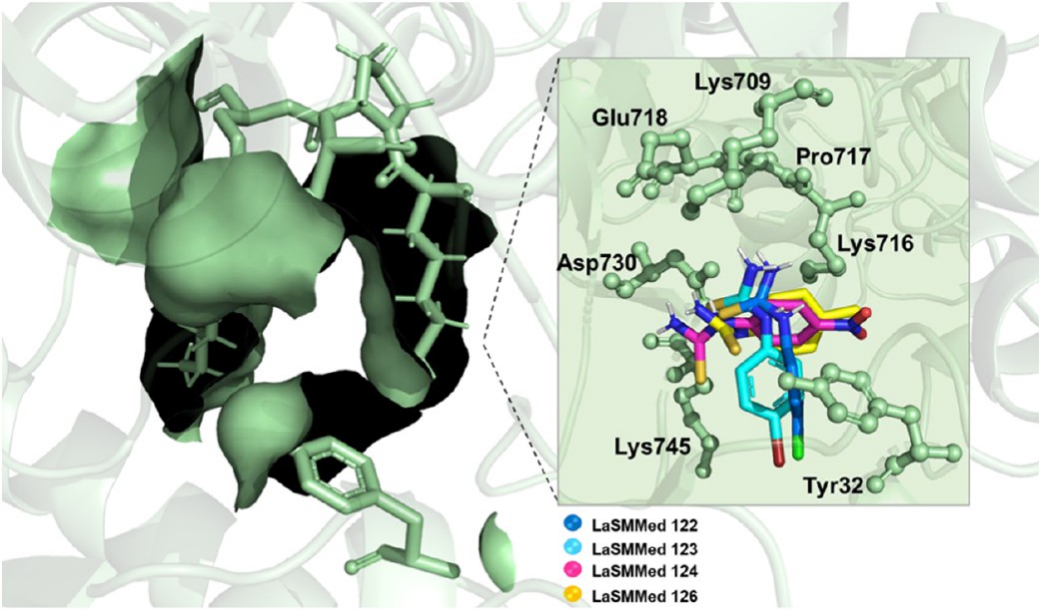
Allosteric site cavity formed by residues Tyr32, Lys709, Lys716, Pro717, Glu718, Asp730, and Lys745 to interactions with **LaSMMed 122–124** and **LaSMMed 126**.

Furthermore, the charged amino groups of lysine and carboxyl groups of aspartate and glutamate provide excellent conditions for H-bond interactions with the thiourea groups of the derivatives. Collectively, these features form a criteria binding pocket that is highly suitable for complexing these derivatives with the urease (Figure 14).

## 3 Conclusions

In summary, six thiourea derivatives derived from cinnamic acid (**LaSMMed 37–46**) were identified through virtual screening as potential urease inhibitors. These compounds interacted with key residues in the active site of HPU, including Ni^2+^ ions and histidine residues, which are crucial for enzymatic activity. The most promising compound, **LaSMMed 42**, showed an inhibition rate against CEU, comparable to that of the standard thiourea. A new series of arylthioureas (**LaSMMed 122–126**) was designed and synthesized based on the structure of **LaSMMed 42**. These compounds exhibited a strong urease inhibitory activity. The arylthioureas demonstrated mixed or competitive inhibition mechanisms. The nitro-substituted derivative, **LaSMMed 124**, showed the highest inhibitory potency.

Molecular dynamics simulations revealed that the arylthioureas preferentially bind to the allosteric site of CEU, forming stable interactions with key residues, such as Tyr32, Lys716, and Glu718. These interactions were consistent with the mixed inhibition mechanism observed *in vitro*. The competitive inhibitor **LaSMMed 125** interacted with the active site residues, including Ni^2+^ ions, while the other arylthioureas showed mixed inhibition by binding to the allosteric site. The primary thiourea subunit was essential for binding to the enzyme’s active site, particularly interacting with Ni^2+^ ions and key amino acid residues. The presence of electron-donating or -withdrawing groups on the aryl ring influenced the inhibitory potency, with the nitro group (**LaSMMed 124**) providing the highest activity. Future studies might investigate further structural modifications, such as adding substituents at positions 2 and 3 of arylthioureas, to enhance the potency of these compounds.

The study successfully identified a series of thiourea and arylthiourea derivatives as potent urease inhibitors. The most effective compounds, particularly **LaSMMed 42** and arylthioureas **LaSMMed 122–126**, showed promising inhibitory activity against urease. The molecular docking and dynamics simulations provided insights into the binding mechanisms and structural requirements for effective urease inhibition, supporting the potential development of these compounds as novel therapeutic agents against urease-producing pathogens.

## 4 Experimental Section

### 4.1 General

Analytical-grade solvents were used as received. For synthetic purposes, solvents and reagents underwent meticulous treatment, distillation, and drying, tailored to the specific requirements outlined in the adopted methodologies and adhering to the procedures elucidated by Armarego and Chai (2003).^28^ NMR spectra were obtained

on a Bruker spectrometer (Model Ascend 400) operating at 400.13 MHz for ^1^H and 100.13 MHz for ^13^C equipped with a 5 mm broadband probe. Deuterated solvents, such as chloroform (CDCl_3_, 7.26 ppm) or dimethyl sulfoxide (DMSO-*d*_6_, 2.50 ppm), were employed for recording the spectra. Signal multiplicities were denoted as singlet (*s*), doublet (*d*), triplet (*t*), quartet (*q*), double doublet (*dd*), double double–doublet (*ddd*), and multiplet (*m*), while coupling constants (*J*) were expressed in Hz. The spectroscopic data for the synthesized substances are detailed in the Supporting Information.

### 4.2 Synthesis of Arylthioureas and Cinnamic Acid Derivatives in the LaSMMed Chemical Library

#### 4.2.1 General Procedure for the Synthesis of Cinnamic Acid Derivatives (LaSMMed 37–45)

Cinnamic acid derivatives were synthesized using the methodology outlined by Brito et al. (2015), with pertinent modifications.^21^ In a 500 mL round-bottom flask, a solution of cinnamic acid (100 mmol) dissolved in 250 mL of dichloromethane, 1.0 mL of dimethylformamide, and 125 mmol of SOCl_2_ was combined. The reaction mixture was then refluxed and stirred for 3 h. Subsequently, the solvent was evaporated under reduced pressure, yielding cinnamoyl chloride as an orange solid product. In the same reaction flask employed for chloride formation, 200 mL of dried acetone was added, along with 100 mmol of NH_4_SCN (1.0 equiv concerning cinnamic acid). The reaction continued under stirring and reflux for 15 min, resulting in the *in situ* formation of cinnamoyl thiocyanate. A 10 mL aliquot of the obtained thiocyanate was dissolved in 10 mL of dry acetone, and with stirring at room temperature, the corresponding amine (10 mmol, 1.0 equiv) was gradually introduced to form the **LaSMMed 37–46** derivatives. The reaction mixture was stirred at room temperature for approximately 40 min. Afterward, the reaction mixture was poured onto crushed ice, and the resulting precipitate was filtered under vacuum. **LaSMMed 37–46** were obtained in yields ranging from 62 to 90% after recrystallization in ethanol.

#### 4.2.2 General Procedure for the Synthesis of Arylthioureas (LaSMMed 122–126)

The synthesis of arylthioureas (**LaSMMed 122–126**) adhered to the procedure outlined by Kataria and collaborators with specific modifications.^29^ A 25 mL round-bottom flask was charged with the corresponding aniline (16 mmol, 1.0 equiv). To this, 5 mL of concentrated hydrochloric acid was gradually added while continuously stirring at room temperature.. The reaction was allowed to proceed under these conditions for approximately 5 to 10 min until a visible change occurred in the reaction medium. At this point, a solution of ammonium thiocyanate (1.5 equiv), previously dissolved in 6 mL of distilled water, was added to the flask. The reaction mixture was stirred at room temperature for an additional 15 min until a noticeable change occurred. After this period, the mixture was heated to a temperature between 70 and 80 °C. Within approximately 30 min, the solid in the medium dissolved, and after several more hours, a color change was observed. Finally, the reaction mixture was then stirred and heated for an additional 24 to 26 h. Afterward, the reaction mixture was poured onto crushed ice, and the resulting precipitate was filtered under vacuum. The products were purified by washing with methanol (2 × 10 mL), resulting in arylthioureas **LaSMMed 122–126** with yields ranging from 38 to 48%.

### 4.3 Molecular Modeling

#### 4.3.1 Preparation of Three-Dimensional Structures of Urease

Crystallographic structures of ureases from C. ensiformis and H. pylori, identified by the respective PDB codes 4H9M and 6ZJA, were obtained from the RCSB Protein Data Bank. These structures, featuring resolutions of 1.52 and 2.00 Å, included cocrystallized ligands: acetohydroxamic acid (HAE) for CEU and the inhibitor 2-{[1-(3,5-dimethylphenyl)-1H-imidazol-2-yl]­sulfanyl}-*N*-hydroxyacetamide (DJM) for HPU. Before the virtual screening, these structures were processed in Discovery Studio Visualizer software (v.21.1.0.20298). In the case of CEU, water (HOH) and 1,2-ethanediol (EDO) molecules were removed, while for HPU, water molecules and noncontributing tertiary chains were excluded. The quaternary structure of the enzyme was further reduced to five chains (A, B, Q, N, and R) to optimize computational efficiency.

#### 4.3.2 Preparation of Ligands

The three-dimensional structures of the molecules were constructed using Discovery Studio Visualizer (v.19.1.0.18287)^30^ and subjected to preliminary energy minimization by molecular mechanics (MMFF) through the “Clean Geometry” command. The protonation state of structures at pH 7.4 was verified using MarvinSketch (v.19.21.0), and most species charges were applied to the correct structures from Discovery Studio Visualizer. For additional structural optimization, ligands underwent energy minimization by the semiempirical method PM7, utilizing the MOPAC2016 program through the Mercury program interface (2020.2.0). All substances from the LaSMMed chemical library used in the virtual screening process are listed in Supporting Information.

#### 4.3.3 Molecular Docking

##### 4.3.3.1 Helicobacter pylori

The three-dimensional structure of urease, obtained through electron microscopy, was prepared for GOLD Suite program calculations (v. 2021.3.0).^31^ Like CEU, the structure was adjusted by removing the cocrystallized ligand and adding hydrogen atoms.

The center of the active site radius was determined as the midpoint between the nickel atoms [Ni(601) and Ni(602)], with coordinates of X = 225.1450, Y = 246.0130, and *Z* = 196.5110, and a radius of 15 Å. Molecular docking of the ligand was carried out in flexible mode for the ligand and rigid mode for the protein, generating 50 docking poses for DJM. The four scoring functions available in the GOLD program (GoldScore, ChemScore, CHEMPLP, and ASP) were employed, utilizing default program settings for calculations.

The docking methodology applied to the ligands was validated through redocking using the ASP scoring function, 50 docking poses, a radius of 15 Å, and the midpoint between the Ni ions as the center. Interactions were analyzed using Discovery Studio Visualizer (v.21.1.0.20298), and figures were generated with Pymol (v2.0.4).^32^ After validation, the same parameters were applied to ligands from the LaSMMed chemical library.

##### 4.3.3.2 Canavalia ensiformis

The molecular modeling study was conducted using the GOLD Suite program (v.2020.1.0, with subsequent studies using v.2022.2.0). The crystallographic structure of urease was prepared for calculations within the software interface by eliminating the cocrystallized ligand and adding hydrogen atoms.

To molecular docking in the active site was applied ligand **LaSMMed 125**, the center of the radius was defined as the midpoint between the nickel atoms [Ni(901) and Ni(902)], with coordinates *X* = 18.7825, *Y* = −57.8095, and *Z* = −24.1515.^18^ For cocrystallized acetohydroxamic acid (**HAE**), 50 docking poses were generated using the four scoring functions available (GoldScore, ChemScore, CHEMPLP, and ASP), employing default program settings for calculations, considering the one with lower root mean square deviation (RMSD) <2.00 Å to the protocol. The methodology was then validated by redocking **HAE**, using the ASP scoring function, 50 docking poses, a radius of 15 Å, and the midpoint between the Ni ions as the center.

The molecular docking of compounds **LaSMMed 122**, **123**, **124**, and **126** in the allosteric site was defined by coordinates *x*: 61.8344, *y*: 23.2354, and *z*: 89.2500 after the FTMap server analysis (https://ftmap.bu.edu/) reported in our previous work^25^ using the ASP scoring function, 50 docking poses, and a radius of 13 Å. Ligands **LaSMMed 122**, **123**, and **124** were docked in the allosteric site without the inhibitor hydroxamic acid (**HAE**) on the active site (free-site). In contrast, **LaSMMed 126** was docked in the allosteric site with **HAE** within (occupied site) and out of the active site.

Molecular docking was conducted in the rigid mode for the protein and the flexible mode for the ligands. The ligand–protein complexes were analyzed by examining interactions using the Discovery Studio Visualizer program (v.19.1.0.18287). Figures were generated using the Pymol program (1.7.4.5 Edu).^32^

#### 4.3.4 Molecular Dynamics

The poses obtained in the CEU docking studies in the active or allosteric sites were used for molecular dynamics simulations with the CHARMM36 force field^33^ employing the GROMACS 2021 program^34^ following the protocol established by our research group.^18^ The protonation state was determined for CEU, considering the pH of 7.4 determined using the H^+2^ server.^35^ Each protein–ligand system was inserted and centered in a triclinic box (dimensions: 13.072 × 9.617 × 8.975 nm; volume: 1128.25 nm^3^) with periodic conditions. The water model considered was TIP3P,^36^ and the complex (target–ligand–water) was neutralized with 19 atoms of Na^+^ ions. Ligand parameters were retrieved from the CGenFF server by uploading the mol2 files with the server’s default settings, and these parameters were incorporated into the protein chain. We obtained the str format from these data, and a script was used to generate the itp and prm files, which are crucial for establishing the topology of the ligands.^37^ The energy minimization, equilibration, and production steps were executed according to our previous work.^18,38^ The energy minimization process was achieved first with the steepest-descent algorithm and then with the gradient conjugate algorithm, applying convergence criteria of 1000 and 100 kJ mol^–1^.nm^–1^, respectively. Next, the equilibration step was performed at 300 K and 1 bar with positional restraints applied to the entire system, except for ions and water molecules. In the first step (1 ns), the NVT ensemble was kept constant, and in the second step (1 ns), the system was considered as an isothermal–isobaric (NPT) ensemble. This equilibration enables atomic speeds that align with the target temperature and pressure. Constraining the protein–ligand atoms also helps ions and water molecules optimally organize along the protein’s surface, creating structured solvation layers. Temperature control was achieved with the V-rescale thermostat^39^ and pressure control through the Parrinello–Rahman barostat.^40^ All bonds to hydrogen atoms in the complex were constrained using the linear constrained solver (LINCS) algorithm.^41^ The long-distance electrostatic interactions were treated using the Particle-Mesh Ewald (PME) algorithm,^42^ and the cutoff radius applied to the van der Waals and Coulomb interactions was 1 nm. Following equilibration, production MD simulations were performed for 100 ns within the NPT ensemble, applying no positional restraints, with a 2 fs integration time and a cutoff radius of 10 Å for long-distance interactions. All complexes were evaluated regarding RMSD (gmx_rms), RMSF (gmx_rmsf), and hydrogen bonds (gmx_hbond) with a cutoff radius of 5.0 Å and a cutoff angle of 30 Å, applying the GROMACS 2021 modules. The HbMap2Grace software^43^ was used to calculate the frequency of the hydrogen bonds, and the VMD software^44^ was used to visualize the trajectories of the simulations. The binding free energies (Δ*G*_bind_) for 100 ns of molecular simulations were calculated using the Linear Interaction Energy (LIE) method with standard coefficients α = 0.161 and β = 0.50 ^27^ through the module added to GROMACS 5.1.4.^45^ We conducted a conformational cluster analysis using the gmx_cluster module from GROMACS 2021 to select 20 thermodynamically appropriate 20 frames for binding free energy calculations. The reference configuration was chosen from the most populated cluster, which reflects the structurally dominant conformation.

### 4.4 Enzyme Assays

#### 4.4.1 Enzyme Inhibition of Urease from C. ensiformis

The *in vitro* inhibitory activity of C. ensiformis urease was conducted using the indophenol reaction, also known as the Berthlot Reaction.^46^ Commercially purchased urease from Merck (Sigma-Aldrich), Jack bean type III (CAS 9002–13–5), was utilized, and the methodology was adapted from Khan et al. (2017).

The assays were performed in triplicate on a 96-well plate, as described by our research group.^18,19^ The procedure involved adding 100 μL of urea (10 mM), 55.0 μL of sodium phosphate buffer solution (100 mM, pH 7.4) supplemented with EDTA (1 mM), and 10 μL of the test substance at 2.00 mM for inhibition percentage assays, previously dissolved in methanol. Subsequently, 25.0 μL of urease enzyme solution (0.0035 mM) was added, and the reaction mixture was incubated at 45–50 °C for 30 min. After incubation, 40.0 μL of phenol reagent (1% phenol and 0.05% sodium nitroprusside) and 40.0 μL of alkaline reagent (1.0% NaOH and 0.05% sodium nitroprusside) were added.^47^ The reaction rested for 15 min, and the absorbance was read at 630 nm using a Loccus model LMR-96i UV–visible microplate reader.

The inhibition percentage (%I) was calculated using the following formula:

$$\%I=100-(\frac{{Abs}_{sample}}{{Abs}_{control}}\times 100)$$

Abs_sample_ is the absorbance observed for the samples, and Abs_control_ is the absorbance observed for the positive control. Thiourea (2.00 mM) served as the standard inhibitor.

To determine half of the maximum inhibitory concentration (IC_50_), substances **LaSMMed 42**, **LaSMMed 122–126**, and the thiourea control were tested at different concentrations (0.2, 0.4, 0.8, 1.0, 1.4, and 2.0 mM). The IC_50_ values were calculated using GraphPad Prism 8.0.2, employing a dose–response curve.

#### 4.4.2 Enzyme Kinetics

Enzyme kinetics were studied following previous work by our research group.^18,19^ The same reaction mixture was applied to determine inhibitory activity, and the IC_50_ was adopted to study enzyme kinetics in C. ensiformis urease. However, only two concentrations of the test substance were used (0.50 and 0.25 mM), along with five different concentrations of urea substrate (0.66, 1.00, 1.50, 2.00, and 2.50 mM) for each sample. The absorbance of the reaction mixture was recorded at 630 nm using a Loccus model LMR-96i UV–visible microplate reader. Michaelis–Menten (nonlinear regression) and Lineweaver–Burk (linear regression) plots, as well as the determination of inhibition constants (*K*_*i*_), *K*_m_, α, and *V*_max_, were made using GraphPad Prism software (version 8.0.2). Enzymatic activity was calculated using an ammonium chloride calibration curve. One unit of enzymatic activity (*U*) is the amount of enzyme capable of releasing 1 μmol of ammonia per minute of reaction (*U* = 1 μmol NH_4_^+^ min^–1^ μmol enzyme^–1^). The assays were performed in triplicate.

### 4.5 Statistical Analysis

Statistical analyses were conducted using the SISVAR program (version 5.8).^48^ Different letters indicate a significant difference between treatments using the Scott–Knott test (*P* < 0.05).

## Supplementary Material



## Notes

Identifier of the compounds from LaSMMed chemical library; Interactions observed in the molecular docking with HPU; NMR spectra of the synthesized substances (PDF)
