# Supplementary material for: Molecular Modeling and In Vitro Evaluation of Thioureas and Arylthioureas as Urease Inhibitors
Source: ACS Omega. 2025 May 22;10(21):21795–812. doi: 10.1021/acsomega.5c01648 (PMC12138827; doi:10.1021/acsomega.5c01648)
Supplement: Supplementary file 1 [file ao5c01648_si_001.pdf]

--- Supplementary Material---

Molecular Modeling and *In vitro* Evaluation of Thioureas and Arylthioureas as Urease Inhibitors

Marciéli Fabris[1], Priscila G. Camargo[2], Mariana L. Silva[1], Camilo H. S. Lima[3], Magaly G. Albuquerque[3], Carlos R. Rodrigues[2], Nailton M. Nascimento-Júnior[4], Marcelle L. F. Bispo[1*]

1. Laboratório de Síntese de Moléculas Medicinais (LaSMMed), Departamento de Química, Universidade Estadual de Londrina (UEL), Rodovia Celso Garcia Cid, PR-445, Km 380, 86057-970, Londrina, Paraná, Brasil.
2. Faculdade de Farmácia, Departamento de Fármacos e Medicamentos, Universidade Federal do Rio de Janeiro, Av. Carlos Chagas Filho, 373, 21941-170, Rio de Janeiro, Rio de Janeiro, Brasil
3. Laboratório de Modelagem Molecular (LabMMol), Instituto de Química, Universidade Federal do Rio de Janeiro, Avenida Athos da Silveira Ramos, nº 149, 21941-909, Rio de Janeiro, Rio de Janeiro, Brasil
4. Laboratório de Química Medicinal, Síntese Orgânica e Modelagem Molecular (LaQMedSOMM), Departamento de Química e Bioquímica, Instituto de Química, Universidade Estadual Paulista (UNESP), Rua Prof. Francisco Degni, 55, 14800-060, Araraquara, São Paulo, Brasil.

Table S1. Compounds of LaSMMed chemical library used in virtual screening

LaSMMed ID	SMILES	Class
1	S=C(N1)N[C@@H](CC(C)C)C1=O	Thiohydantoin
2	S=C(N1)N[C@@H](CC2=CNC3=C2C=CC=C3)C1=O	Thiohydantoin
3	S=C(N1)N[C@@H](CC2=CC=CC=C2)C1=O	Thiohydantoin
4	S=C(N1)N[C@@H](CC2=CC=CC=C2)C1=O	Thiohydantoin
5	S=C(N1)N2[C@@H](CCC2)C1=O	Thiohydantoin
6	S=C(N1)N[C@@H](C(C)C)C1=O	Thiohydantoin
7	S=C(N1)N[C@@H](CCSC)C1=O	Thiohydantoin
8	S=C(NC/1=O)NC1=C/C	Thiohydantoin
9	S=C(N1)N(C(C)=O)CC1=O	Thiohydantoin
10	S=C(N1)NCC1=O	Thiohydantoin
11	S=C(N1)N([C@@H](C)C1=O)C(C)=O	Thiohydantoin
12	S=C(N1)N[C@@H](C)C1=O	Thiohydantoin
13	S=C(N1)N[C@@H](CC2=CC=C(O)C=C2)C1=O	Thiohydantoin
14	S=C(N1)N([C@@H](CC(N)=O)C1=O)C(C)=O	Thiohydantoin
15	S=C(N1)N[C@@H](CC(N)=O)C1=O	Thiohydantoin
16	S=C(N1)N([C@@H](CCC(N)=O)C1=O)C(C)=O	Thiohydantoin
17	S=C(N1)N[C@@H](CC2=CNC=N2)C1=O	Thiohydantoin
18	S=C(N1)N([C@@H](CC2=CNC3=C2C=CC=C3)C1=O)C(C)=O	Thiohydantoin
19	S=C(N1)N([C@@H](CC2=CC=CC=C2)C1=O)C(C)=O	Thiohydantoin
20	S=C(N1)N([C@@H](C(C)C)C1=O)C(C)=O	Thiohydantoin
21	S=C(N1)N(C(C)=O)[C@@H](CC(C)C)C1=O	Thiohydantoin
22	S=C(N1)N(C(C)=O)[C@@H](CCSC)C1=O	Thiohydantoin
23	O=C(N1)N[C@@H](C(C)C)C1=O	Hydantoin
24	O=C(N1)N[C@@H](CC(C)C)C1=O	Hydantoin
25	O=C(N1)N[C@@H](CCSC)C1=O	Hydantoin
26	O=C(N1)N[C@@H](CC2=CC=CC=C2)C1=O	Hydantoin
27	O=C(N1)N[C@@H](CC2=CC=C(O)C=C2)C1=O	Hydantoin
28	O=C(N1)N[C@@H](CC2=CNC3=C2C=CC=C3)C1=O	Hydantoin
29	O=C(CN1)NC1=O	Hydantoin
30	O=C(/C(N1)=C/C)NC1=O	Hydantoin
31	O=C([C@H](CCC(O)=O)N1)NC1=O	Hydantoin
32	O=C([C@H](C)N1)NC1=O	Hydantoin
33	O=C([C@H]1N2CCC1)NC2=O	Hydantoin
34	O=C(N1)N[C@@H](CC2=CNC=N2)C1=O	Hydantoin
35	S=C(N1)N(C(C)=O)[C@@H](CCC(N)=O)C1=O	Hydantoin
36	O=C([C@H](CC(O)=O)N1)NC1=O	Hydantoin
37	O=C(NC(NC)=S)/C=C/C1=CC=CC=C1	Cinnamoylthiourea 203
38	O=C(NC(NCC)=S)/C=C/C1=CC=CC=C1	Cinnamoylthiourea 204
39	O=C(NC(NCCCC)=S)/C=C/C1=CC=CC=C1	Cinnamoylthiourea 205
40	O=C(NC(NCCCCCC)=S)/C=C/C1=CC=CC=C1	Cinnamoylthiourea 206
41	O=C(NC(NC(C)C)=S)/C=C/C1=CC=CC=C1	Cinnamoylthiourea 207
42	O=C(NC(N)=S)/C=C/C1=CC=CC=C1	Cinnamoylthiourea 208
43	O=C(NC(NC1=CC=CC=C1)=S)/C=C/C2=CC=CC=C2	Cinnamoylthiourea 209
44	O=C(NC(NC1=CC=C(OC)C=C1)=S)/C=C/C2=CC=CC=C2	Cinnamoylthiourea 210
45	O=C(NC(NC1=CC=C(F)C=C1)=S)/C=C/C2=CC=CC=C2	Cinnamoylthiourea 211
46	O=C(NC(NC1=CC=C(C)C=C1)=S)/C=C/C2=CC=CC=C2	Cinnamoylthiourea 212
47	O=C(OC1=CC(C(C)C)=CC=C1C)C2=CC=CC=C2	Carvacrol
48	O=C(OC1=CC(C(C)C)=CC=C1C)/C=C/C2=CC=CC=C2	Carvacrol
49	O=C(OC1=CC(C(C)C)=CC=C1C)C2=CC=CC=C2Cl	Carvacrol
50	O=C(OC1=CC(C(C)C)=CC=C1C)C2=CC=CC(Cl)=C2	Carvacrol
51	O=C(OC1=CC(C(C)C)=CC=C1C)C2=CC=C(Cl)C=C2	Carvacrol
52	O=C(OC1=CC(C(C)C)=CC=C1C)C2=CC=CC=C2OC	Carvacrol
53	O=C(OC1=CC(C(C)C)=CC=C1C)C2=CC=CC(OC)=C2	Carvacrol
54	O=C(OC1=CC(C(C)C)=CC=C1C)C2=CC=C(OC)C=C2	Carvacrol

55	O=C(OC1=CC(C(C)C)=CC=C1C)C2=CC=CC=C2[N+]([O-])=O	Carvacrol
56	O=C(OC1=CC(C(C)C)=CC=C1C)C2=CC=CC([N+]([O-])=O)=C2	Carvacrol
57	O=C(OC1=CC(C(C)C)=CC=C1C)C2=CC=C([N+]([O-])=O)C=C2	Carvacrol
58	O=C1OC2=CC=CC=C2C=C1C(NC(NC3=CC=C(Cl)C=C3)=S)=O	Coumarylthiourea
59	O=C1C(C(NC2=CC=CC=C2)=O)=CC3=C(C=CC=C3)O1	Coumarilamide
60	O=C1C(C(NC2=C(Br)C=CC=C2)=O)=CC3=C(C=CC=C3)O1	Coumarilamide
61	O=C1C(C(NC2=CC(Br)=CC=C2)=O)=CC3=C(C=CC=C3)O1	Coumarilamide
62	O=C1C(C(NC2=CC=C(Br)C=C2)=O)=CC3=C(C=CC=C3)O1	Coumarilamide
63	O=C1C(C(NC2=C([N+]([O-])=O)C=CC=C2)=O)=CC3=C(C=CC=C3)O1	Coumarilamide
64	O=C1C(C(NC2=CC([N+]([O-])=O)=CC=C2)=O)=CC3=C(C=CC=C3)O1	Coumarilamide
65	O=C1C(C(NC2=CC=C([N+]([O-])=O)C=C2)=O)=CC3=C(C=CC=C3)O1	Coumarilamide
66	O=C1C(C(NC2=C(Cl)C=CC=C2)=O)=CC3=C(C=CC=C3)O1	Coumarilamide
67	O=C1C(C(NC2=CC(Cl)=CC=C2)=O)=CC3=C(C=CC=C3)O1	Coumarilamide
68	O=C1C(C(NC2=CC=C(Cl)C=C2)=O)=CC3=C(C=CC=C3)O1	Coumarilamide
69	CC1=CC2=CC=CC=C2N1	Indole
70	CC(C)CC(N1)=CC2=C1C=CC=C2	Indole
71	C12=CC=CC=C1NC(C3=CC=CC=C3)=C2	Indole
72	O=C(O)C(C=C1)=CC=C1C2=CC3=CC=CC=C3N2	Indole
73	COC(C=C1)=CC=C1C2=CC3=CC=CC=C3N2	Indole
74	BrC(C=C1)=CC=C1C2=CC3=CC=CC=C3N2	Indole
75	O=C(OC)C1=CC=C(C(N2)=CC3=C2C=CC=C3)C=C1	Indole
76	O=C(N1C(C2=CC=CC=C2)=CC3=C1C=CC=C3)OC(C)(C)C	Indole
77	O=C(N1C(CC(C)C)=CC2=C1C=CC=C2)C3=CC=CC=C3	Indole
78	ClC1=CC2=C(NC(C3=CC=CC=C3)=C2)C=C1	Indole
79	COC(C=C1)=CC=C1C2=CC3=CC(Cl)=CC=C3N2	Indole
80	BrC(C=C1)=CC=C1C2=CC3=CC(Cl)=CC=C3N2	Indole
81	O=C(O)C(C=C1)=CC=C1C2=CC3=CC(Cl)=CC=C3N2	Indole
82	O=C(OC)C1=CC=C(C(N2)=CC3=C2C=CC(Cl)=C3)C=C1	Indole
83	O=C1OC[C@H](CC(C)C)N1C(C2=CC=CC=C2)=O	Oxazolidinone
84	O=C1OC[C@H](CC2=CC=CC=C2)N1C(C3=CC=CC=C3)=O	Oxazolidinone
85	O=C1OC[C@H](CC(C)C)N1C(C2=CC=C(Cl)C=C2)=O	Oxazolidinone
86	O=C1OC[C@H](CC2=CC=CC=C2)N1C(C3=CC=C(Cl)C=C3)=O	Oxazolidinone
87	O=C1OC[C@H](CC(C)C)N1C(C2=CC=C(OC)C=C2)=O	Oxazolidinone
88	O=C1OC[C@H](CC2=CC=CC=C2)N1C(C3=CC=C(OC)C=C3)=O	Oxazolidinone
89	O=S(C1=CC=C(C)C=C1)(NN2C(C3=CC=CC=C3)SCC2=O)=O	Thiazolidinone
90	O=S(C1=CC=C(C)C=C1)(NN2C(C3=CC=C(OC)C=C3)SCC2=O)=O	Thiazolidinone
91	O=S(C1=CC=C(C)C=C1)(NN2C(C3=CC=C(N(C)C)C=C3)SCC2=O)=O	Thiazolidinone
92	O=S(C1=CC=C(C)C=C1)(NN2C(C3=CC=C(C(O)=O)C=C3)SCC2=O)=O	Thiazolidinone
93	O=S(C1=CC=C(C)C=C1)(NN2C(C3=CC=C(C(OC)=O)C=C3)SCC2=O)=O	Thiazolidinone
94	O=S(C1=CC=C(C)C=C1)(NN2C(C3=CC=C(C(OCC)=O)C=C3)SCC2=O)=O	Thiazolidinone
95	O=C1C(C(NCCCC)=O)=CC2=C(C=CC=C2)O1	Cumarilamide
96	O=C1C(C(N(CC)CC)=O)=CC2=C(C=CC=C2)O1	Cumarilamide
97	O=C1C(C(NCCN2CCOCC2)=O)=CC3=C(C=CC=C3)O1	Cumarilamide
98	O=C1C=C(C(NC2=C(Br)C=CC=C2)=O)OC3=CC=CC=C31	Cumarilamide
99	O=C1C=C(C(NC2=C(Cl)C=CC=C2)=O)OC3=CC=CC=C31	Cumarilamide
100	O=C1OCCN1C(C2=CC=CC=C2)=O	Oxazolidinone
101	O=C1OCCN1C(C2=CC=C(Cl)C=C2)=O	Oxazolidinone
102	O=C1OCCN1C(C2=CC=C(OC)C=C2)=O	Oxazolidinone
103	O=C1OC[C@H](C)N1C(C2=CC=CC=C2)=O	Oxazolidinone
104	O=C1OC[C@H](C)N1C(C2=CC=C(Cl)C=C2)=O	Oxazolidinone
105	O=C1OC[C@H](C)N1C(C2=CC=C(OC)C=C2)=O	Oxazolidinone
106	O=C(OCCO)C1=CC=C(O)C(OC)=C1	Apocynin ester analogue
107	O=C(OCCCCCCO)C1=CC=C(O)C(OC)=C1	Apocynin ester analogue
108	O=C(OCCOCCOCCO)C1=CC=C(O)C(OC)=C1	Apocynin ester analogue
109	O=C(OCCCCO)C1=CC=C(O)C(OC)=C1	Apocynin ester analogue
110	CC(C)CC(/C=C/C1=CC=C(O)C(OC)=C1)=O	α,β-unsaturated analogue of apocynin
111	O=C(/C=C/C1=CC=C(O)C(OC)=C1)C2=CC=CC=C2	α,β-unsaturated analogue

		of apocynin
112	CC(/C=C/C1=CC=C(O)C(OC)=C1)=O	α,β-unsaturated analogue of apocynin
113	O=C(/C=C/C1=CC=C(O)C(OC)=C1)C2=CC=C([N+]([O-])=O)C=C2	α,β-unsaturated analogue of apocynin
114	O=C(/C=C/C1=CC=C(O)C(OC)=C1)C2=CC=C(OC)C=C2	α,β-unsaturated analogue of apocynin
115	O=C(/C=C/C1=CC=C(O)C(OC)=C1)C2=CC=C(Br)C=C2	α,β-unsaturated analogue of apocynin
116	O=C1C=C(C(NC2=CC=CC=C2)=O)OC3=CC=CC=C31	Cromone
117	O=C1C=C(C(NC2=CC=C(Cl)C=C2)=O)OC3=CC=CC=C31	Cromone
118	O=C1C=C(C(NC2=CC=C(Br)C=C2)=O)OC3=CC=CC=C31	Cromone
119	FC1=CC2=C(NC(C3=CC=CC=C3)=C2)C=C1	Indole
120	O=C(/C=C/C1=CC=C(O)C(OC)=C1)C2=CC=C(C)C=C2	α,β-unsaturated analogue of apocynin
121	O=C1C=C(C(NC2=CC=C([N+]([O-])=O)C=C2)=O)OC3=CC=CC=C31	Cromone
122	NC(NC1=CC=C(Cl)C=C1)=S	Arylthiourea
123	NC(NC1=CC=C(Br)C=C1)=S	Arylthiourea
124	NC(NC1=CC=C([N+]([O-])=O)C=C1)=S	Arylthiourea
125	NC(NC1=CC=C(OC)C=C1)=S	Arylthiourea
126	NC(NC1=CC=C(C)C=C1)=S	Arylthiourea
127	CC(OC1=CC(C(C)C)=CC=C1C)=O	Carvacrol

Interactions observed in the molecular docking of substances from the LaSMMed library in *Helicobacter pylori* urease

Table S2 – Interactions of LaSMMed 1-8 substances with HPU

	**LMED 1**	**LMED 2**	**LMED 3**	**LMED 4**	**LMED 5**	**LMED 6**	**LMED 7**	**LMED 8**
**Score**	22.83	32.54	25.59	19.86	21.96	25.18	20.53	19.07
**G47R**								
**H136**								
**H138**								
**A169**								
**K219**								
**H221**								
**E222**								
**D223**								
**H248**								
**H274**								
**G279**								
**G280**								
**C321**								
**H322**								
**R338**								
**D362**								
**A365**								
**M366**								
**Ni601**								
**Ni602**								

Color legend according to interaction types:

	H-bonds		π – π Stacking		π – anion
	Non-conventional H-bonds		π-π T- Stacking		π – cation
	Atractive charge (O-+N)		π – alkyl		Sulfur – X (O.N.S)
	Metal – aceptor		Alkyl		Halogen interaction
	π – hidrogen Interaction		π – sulfur		π – σ

Table S3 – Interactions of LaSMMed 9-16 substances with HPU

	LMED 9	LMED 10	LMED 11	LMED 12	LMED 13	LMED 14	LMED 15	LMED 16
**Score**	17.93	19.57	19.43	28.81	23.79	25.46	30.05	28.69
**G47R**								
**H136**								
**H138**								
**A169**								
**K219**								
**H221**								
**E222**								
**D223**								
**H248**								
**H274**								
**G279**								
**G280**								
**H322**								
**R338**								
**D362**								
**A365**								
**Ni601**								
**Ni602**								

Table S4 – Interactions of LaSMMed 17-24 substances with HPU

	LMED 17	LMED 18	LMED 19	LMED 20	LMED 21	LMED 22	LMED 23	LMED 24
**Score**	29.00	27.06	20.27	22.96	21.62	21.62	21.13	22.35
**G47R**								
**H136**								
**H138**								
**A169**								
**K219**								
**H221**								
**D223**								
**H248**								
**H274**								
**G279**								
**M317**								
**L318**								
**C321**								
**H322**								
**R338**								
**D362**								
**A365**								
**M366**								
**Ni601**								
**Ni602**								

Table S5 – Interactions of LaSMMed 25-32 substances with HPU

	LMED 25	LMED 26	LMED 27	LMED 28	LMED 29	LMED 30	LMED 31	LMED 32
Score	24.31	28.45	29.76	17.74	19.02	25.97	19.02	23.52
**G47R**								
**A169**								
**H221**								
**E222**								
**D223**								
**H248**								
**G279**								
**G280**								
**H322**								
**R338**								
**D362**								
**A365**								
**M366**								
**Ni601**								
**Ni602**								

Table S6 – Interactions of LaSMMed 33-38 substances with HPU

	LMED 33	LMED 34	LMED 35	LMED 36	LMED 37	LMED 38
**Score**	27.83	22.46	32.05	33.57	36.29	39.61
**H136**						
**H138**						
**A169**						
**K219**						
**H221**						
**D223**						
**H248**						
**H274**						
**G279**						
**M317**						
**L318**						
**C321**						
**H322**						
**D362**						
**A365**						
**M366**						
**Ni601**						
**Ni602**						

Table S7 – Interactions of LaSMMed 39-44 substances with HPU

	LMED 39	LMED 40	LMED 41	LMED 42	LMED 43	LMED 44	LMED 45
**Score**	33.95	28.10	34.34	39.69	37.37	37.07	37.17
**H136**							
**H138**							
**A169**							
**K219**							
**H221**							
**E222**							
**D223**							
**H248**							
**H274**							
**G279**							
**M317**							
**L318**							
**C321**							
**H322**							
**D362**							
**A365**							
**M366**							
**Ni601**							
**Ni602**							

Table S8 – Interactions of LaSMMed 46-55 substances with HPU

	LMed 46	LMed 47	LMed 48	LMed 49	LMed 50	LMed 51	LMed 52	LMed 53	LMed 54	LMed 55
Score	20.9	29.6	27.9	28.7	30.5	26.6	28.7	29.9	26.7	28.9
**G47(R)**										
**H221**										
**E222**										
**H248**										
**T251**										
**G280**										
**H314**										
**M317**										
**L318**										
**C321**										
**H322**										
**R338**										
**I339**										
**M366**										
**I467**										

Table S9 – Interactions of LaSMMed 56-65 substances with HPU

	LMed 56	LMed 57	LMed 58	LMed 59	LMed 60	LMed 61	LMed 62	LMed 63	LMed 64	LMed 65
Score	30.4	28.1	37.9	30.0	30.3	26.5	25.1	27.2	28.8	27.9
**F45R**										
**G46R**										
**K49R**										
**H136**										
**H138**										
**A169**										
**K219**										
**H221**										
**E222**										
**D223**										
**H248**										
**T251**										
**L252**										
**H274**										
**G279**										
**G280**										
**H314**										
**M317**										
**L318**										
**C321**										
**H322**										
**R338**										
**D362**										
**A365**										
**M366**										
**Ni601**										

**Ni602**										

Table S10 – Interactions of LaSMMed 66-75 substances with HPU

	**LMed 66**	**LMed 67**	**LMed 68**	**LMed 69**	**LMed 70**	**LMed 71**	**LMed 72**	**LMed 73**	**LMed 74**	**LMed 75**
Score	29.0	27.7	26.0	18.9	22.3	24.6	28.6	28.5	24.4	24.9
**F45R**										
**G47R**										
**H138**										
**A169**										
**H221**										
**E222**										
**D223**										
**H248**										
**L252**										
**G280**										
**H314**										
**M317**										
**L318**										
**C321**										
**H322**										
**R338**										
**D362**										
**A365**										
**M366**										
**Ni601**										
**Ni602**										

Table S11 – Interactions of LaSMMed 76-85 substances with HPU

	LMed 76	LMed 77	LMed 78	LMed 49	LMed 80	LMed 81	LMed 82	LMed 83	LMed 84	LMed 85
Score	27.7	26.9	25.1	24.9	20.9	29.6	23.4	24.8	27.2	25.8
**K49R**										
**H138**										
**A169**										
**H221**										
**E222**										
**D223**										
**H248**										
**T251**										
**L252**										
**H314**										
**M317**										
**L318**										
**C321**										
**H322**										
**H323**										
**F334**										
**R338**										
**I339**										
**D362**										
**A365**										
**M366**										
**I467**										
**P468**										
**Ni601**										

**Ni602**										

Table S12 – Interactions of LaSMMed 86-91 substances with HPU

	**LMed 86**	**LMed 87**	**LMed 88**	**LMed 89_R**	**LMed 89_S**	**LMed 90_R**	**LMed 90_S**	**LMed 91_R**	**LMed 91_S**
Score	28.5	25.8	28.5	33.1	30.9	34.4	31.3	35.4	32.4
**A169**									
**H221**									
**E222**									
**D223**									
**H248**									
**G279**									
**G280**									
**H314**									
**M317**									
**L318**									
**C321**									
**H322**									
**R338**									
**I339**									
**A365**									
**M366**									
**I467**									
**Ni601**									
**Ni602**									

Table S13 – Interactions of LaSMMed 92-97 substances with HPU

	LMed 92_R	LMed 92_S	LMed 93_R	LMed 93_S	LMed 94_R	LMed 94_S	LMed 95	LMed 96	LMed 97
Score	30.5	33.8	35.1	33.1	31.8	33.6	28.2	26.0	32.8
**F45R**									
**G47R**									
**K49R**									
**A169**									
**H221**									
**E222**									
**D223**									
**H248**									
**L252**									
**G279**									
**G280**									
**H314**									
**M317**									
**L318**									
**C321**									
**H322**									
**H323**									
**F334**									
**R338**									
**I339**									
**A365**									
**M366**									
**Ni601**									
**Ni602**									

Table S14 – Interactions of LaSMMed 98-107 substances with HPU

	LMed 98	LMed 99	LMed 100	LMed 101	LMed 102	LMed 103	LMed 104	LMed 105	LMed 106	LMed 107
Score	27.16	28.82	22.75	22.02	24.85	22.22	21.77	25.08	28.84	32.29
**G47(R)**										
**H138**										
**A169**										
**K219**										
**H221**										
**E222**										
**D223**										
**H248**										
**T251**										
**H274**										
**A278**										
**G279**										
**G280**										
**M317**										
**L318**										
**C321**										
**H322**										
**R338**										
**I339**										
**D362**										
**A365**										
**M366**										
**Ni601**										
**Ni602**										

Table S15 – Interactions of LaSMMed 108-117 substances with HPU

	LMed 108	LMed 109	LMed 110	LMed 111	LMed 112	LMed 113	LMed 114	LMed 115	LMed 116	LMed 117
Score	35.49	30.47	28.62	33.20	25.71	31.06	29.18	30.44	28.14	27.31
**G47(R)**										
**A169**										
**K219**										
**H221**										
**E222**										
**D223**										
**H248**										
**L252**										
**H274**										
**A278**										
**G279**										
**H314**										
**M317**										
**L318**										
**C321**										
**H322**										
**H323**										
**R338**										
**I339**										
**D362**										
**A365**										
**M366**										
**Ni601**										
**Ni602**										

Table S16 – Interactions of LaSMMed 118-126 substances with HPU

	LMed 118	LMed 119	LMed 120	LMed 121	LMed 122	LMed 123	LMed 124	LMed 125	LMed 126	LMed 127
Score	25.80	26.80	32.97	27.44	22.57	24.41	27.99	27.36	26.08	26.52
**K49(R)**										
**H136**										
**H138**										
**A169**										
**K219**										
**H221**										
**E222**										
**D223**										
**H248**										
**L252**										
**H274**										
**G279**										
**M317**										
**L318**										
**C321**										
**H322**										
**H323**										
**I339**										
**D362**										
**A365**										
**M366**										
**Ni601**										

NMR spectra of the synthesized substances

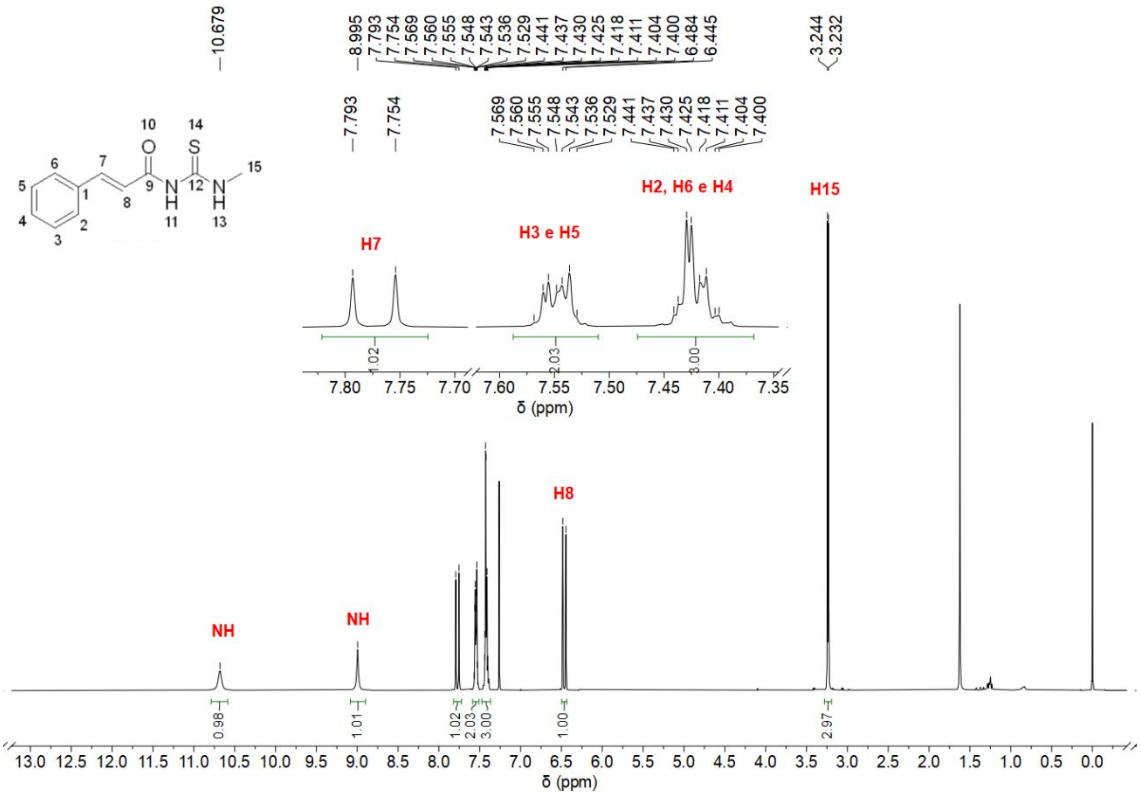

Figure S1 – ^{1}H NMR spectrum (400 MHz, $CDCl_3$) of **LMed 37**

***N*-(methylcarbamothioyl)cinnamamide (LMed 37):** Yellow solid; 69% yield; MP: 215-216 **Lit:** 210-211 °C. NMR ^{1}H [$CDCl_3$, 400 MHz] δ: 10.68 (s, 1H), 9.00 (s, 1H), 7.77 (d, *J* = 15.7 Hz, 1H), 7.59 – 7.51 (m, 2H), 7.47 – 7.37 (m, 3H), 6.46 (d, *J* = 15.7 Hz, 1H), 3.24 (d, *J* = 4.8 Hz, 3H).

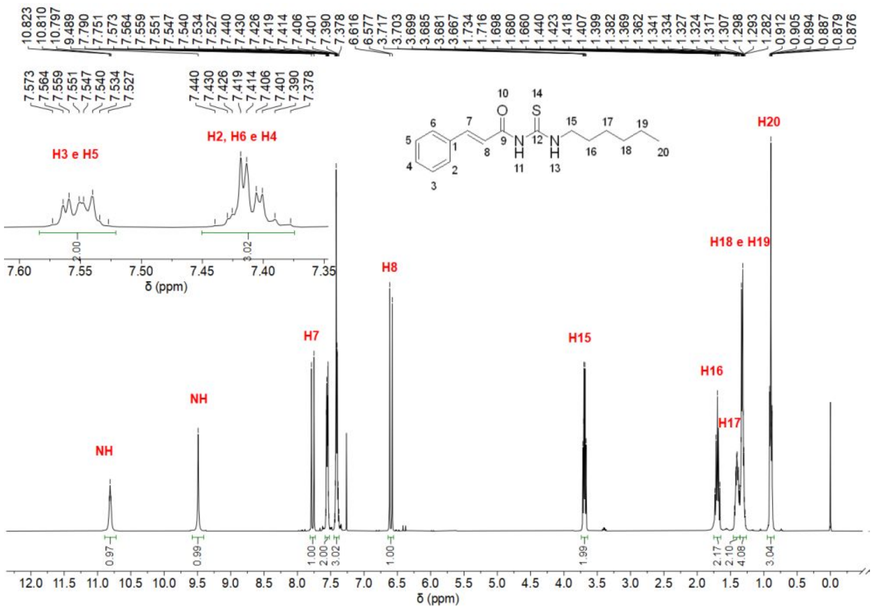

Figure S2 – ^{1}H NMR spectrum (400 MHz, $CDCl_3$) of **LMed 40**

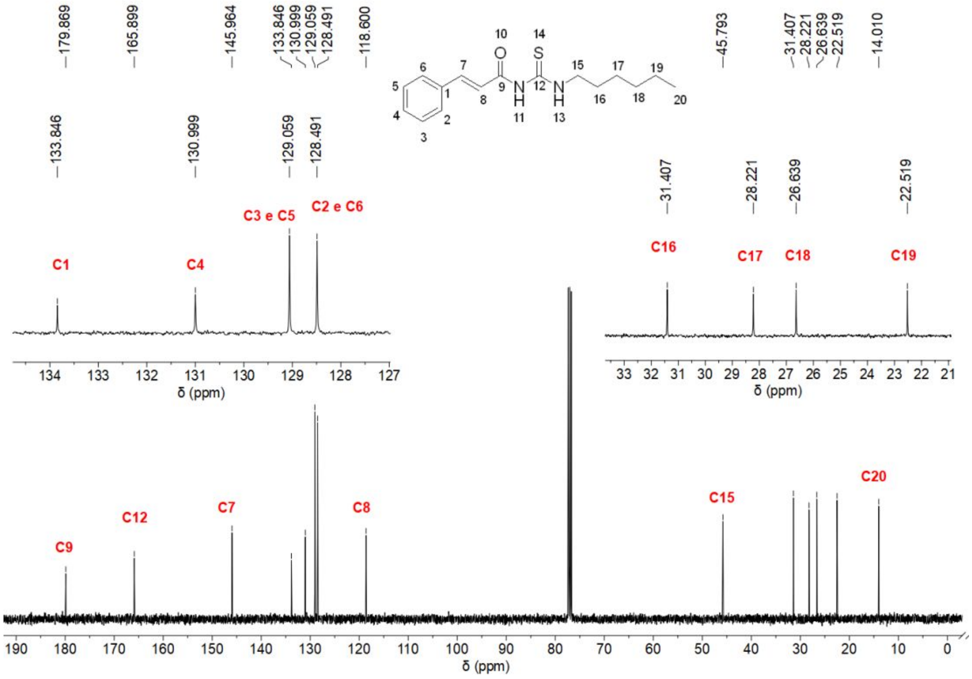

Figure S3 – ^{13}C NMR spectrum (100 MHz, $CDCl_3$) of **LMed 40**

***N*-(hexylcarbamothioyl)cinnamamide (LMed 40):** Yellowish solid; 62% yield. MP: 77 ºC. NMR ^{1}H [$CDCl_3$, 400 MHz] δ: 10.81 (t, *J* = 5.4 Hz, 1H), 9.49 (s, 1H), 7.77 (d, *J* = 15.5 Hz, 1H), 7.55 (qd, *J* = 5.3, 3.2 Hz, 2H), 7.41 (dd, *J* = 5.1, 2.0 Hz, 3H), 6.60 (d, *J* = 15.5 Hz, 1H), 3.69 (td, *J* = 7.2, 5.4 Hz, 2H), 1.70 (p, *J* = 7.9, 7.5 Hz, 2H), 1.46 – 1.36 (m, 2H), 1.36 – 1.26 (m, 4H), 0.95 – 0.85 (m, 3H). NMR ^{13}C [$CDCl_3$, 100 MHz] δ: 179.87, 165.90, 145.96, 133.85, 131.00, 129.06, 128.49, 118.60, 45.79, 31.41, 28.22, 26.64, 22.52, 14.01.

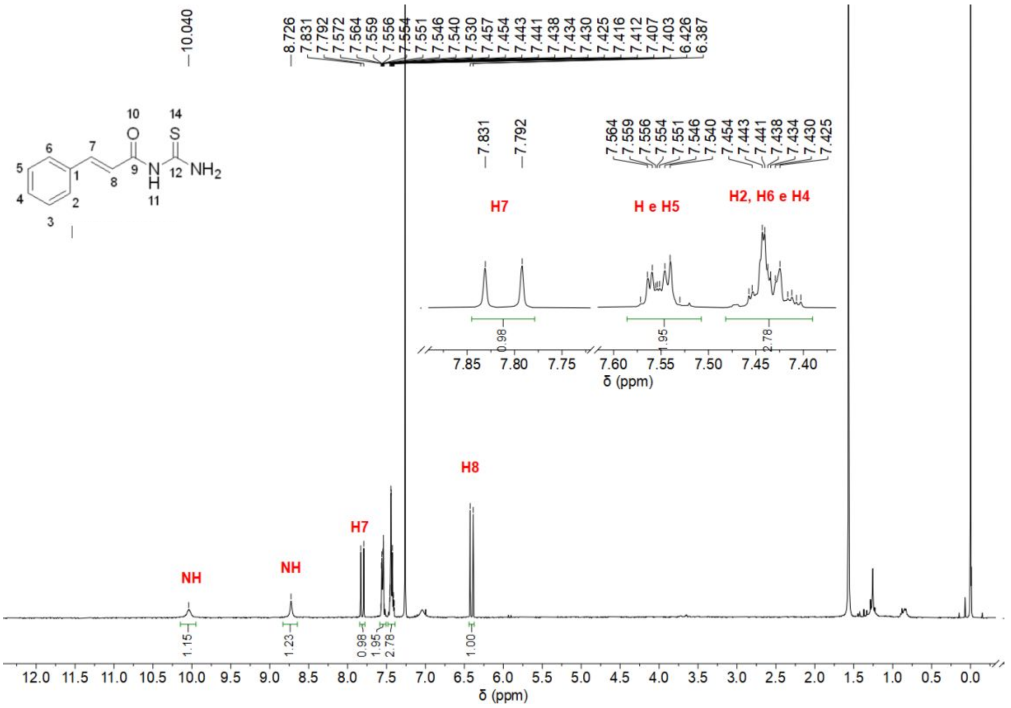

Figure S4 – ^{1}H NMR spectrum (400 MHz, $CDCl_3$) of **LMed 42**

***N*-carbamothioylcinnamamide (LMed 42):** White solid; 74% yield. MP: 217-218 ºC. NMR ^{1}H [$CDCl_3$, 400 MHz] δ: 10.04 (s, 1H), 8.73 (s, 1H), 7.81 (d, J = 15.5 Hz, 1H), 7.59 – 7.51 (m, 2H), 7.48 – 7.39 (m, 3H), 6.41 (d, J = 15.5 Hz, 1H).

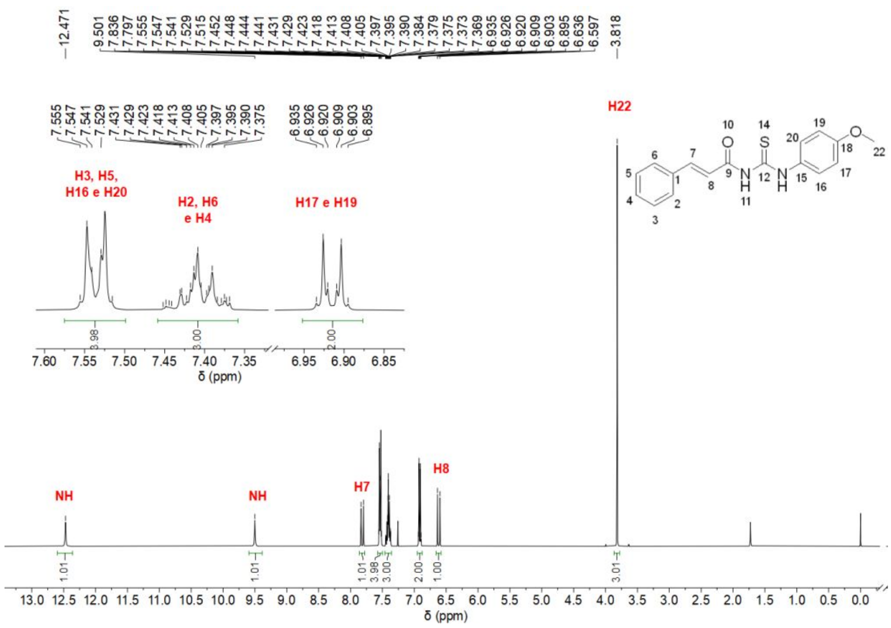

Figure S5 – ^{1}H NMR spectrum (400 MHz, $CDCl_3$) of **LMed 44**

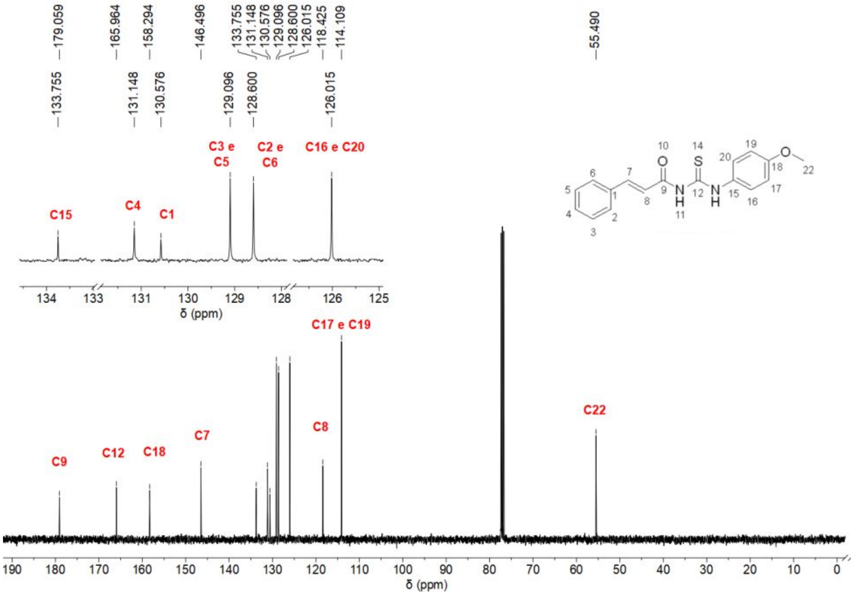

Figure S6– ^{13}C NMR spectrum (100 MHz, $CDCl_3$) of **LMed 44**

***N*-(4-methoxyphenyl)carbamothioyl)cinnamamide (LMed 44):** Green solid; 75% yield; MP. 173-174 ºC **Lit:** 165-167 ºC. NMR ^{1}H [$CDCl_3$, 400 MHz] δ: 12.47 (s, 1H), 9.50 (s, 1H), 7.82 (d, *J* = 15.7 Hz, 1H), 7.58 – 7.50 (m, 4H), 7.46 – 7.36 (m, 3H), 6.95 – 6.88 (m, 2H), 6.62 (d, *J* = 15.5 Hz, 1H), 3.82 (s, 3H). NMR ^{13}C [$CDCl_3$, 100 MHz] δ: 179.06, 165.96, 158.29, 146.50, 133.75, 131.15, 130.58, 129.10, 128.60, 126.02, 118.43, 114.11, 55.49

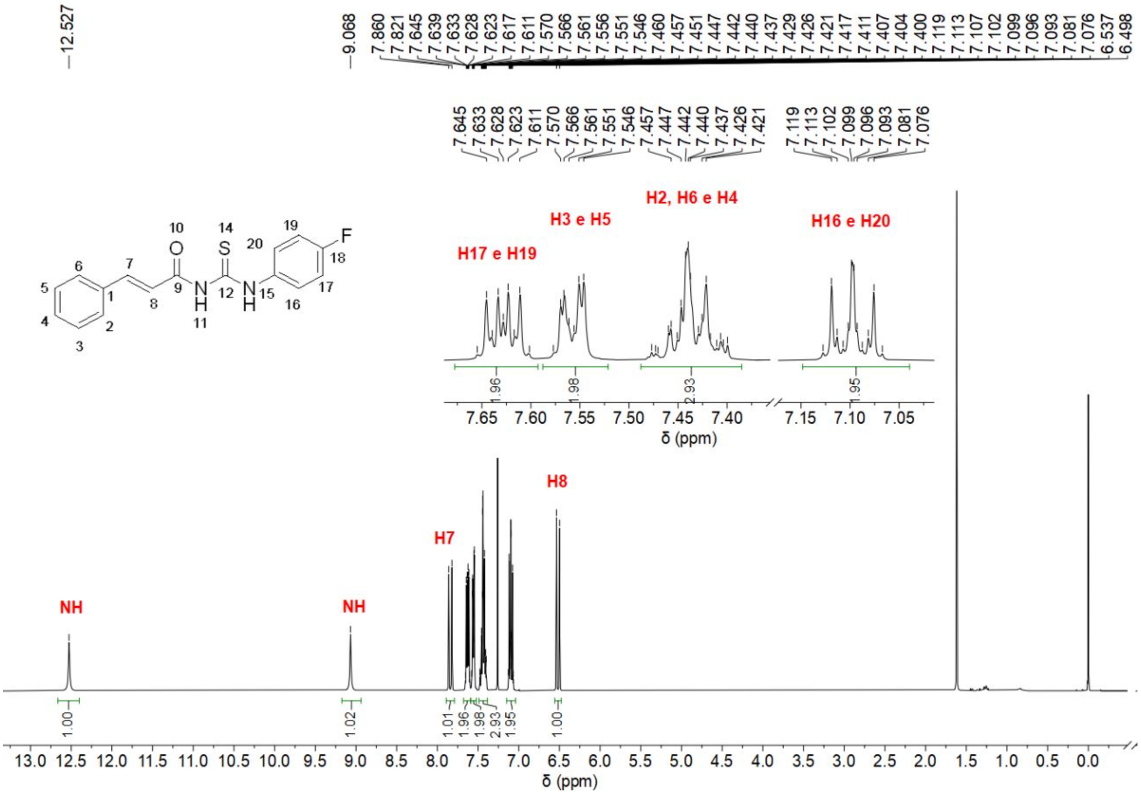

Figure S7 – ^{1}H NMR spectrum (400 MHz, $CDCl_3$) of **LMed 45**

***N*-(4-fluorophenyl)carbamothioyl)cinnamamide (LMed 45):** Yellowish solid; 72% yield. MP: 200-202 ºC. NMR ^{1}H [$CDCl_3$, 400 MHz] δ: 12.53 (s, 1H), 9.07 (s, 1H), 7.84 (d, J = 15.6 Hz, 1H), 7.68 – 7.59 (m, 2H), 7.59 – 7.52 (m, 2H), 7.49 – 7.39 (m, 3H), 7.15 – 7.04 (m, 2H), 6.52 (d, J = 15.7 Hz, 1H).

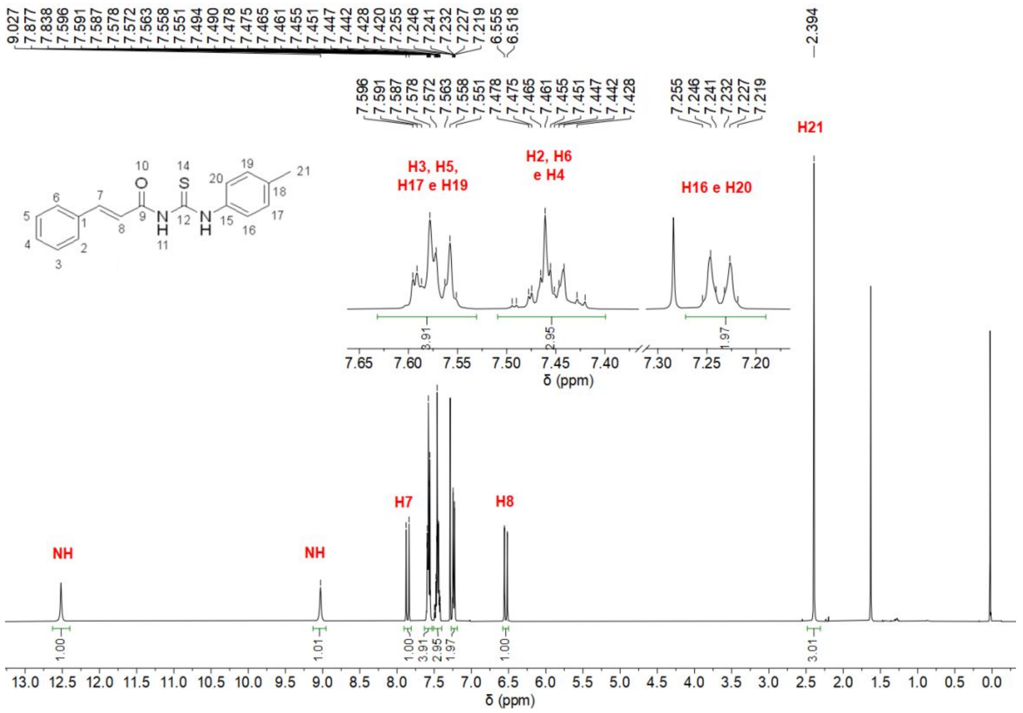

Figure S8 – ^{1}H NMR spectrum (400 MHz, $CDCl_3$) of **LMed 46**

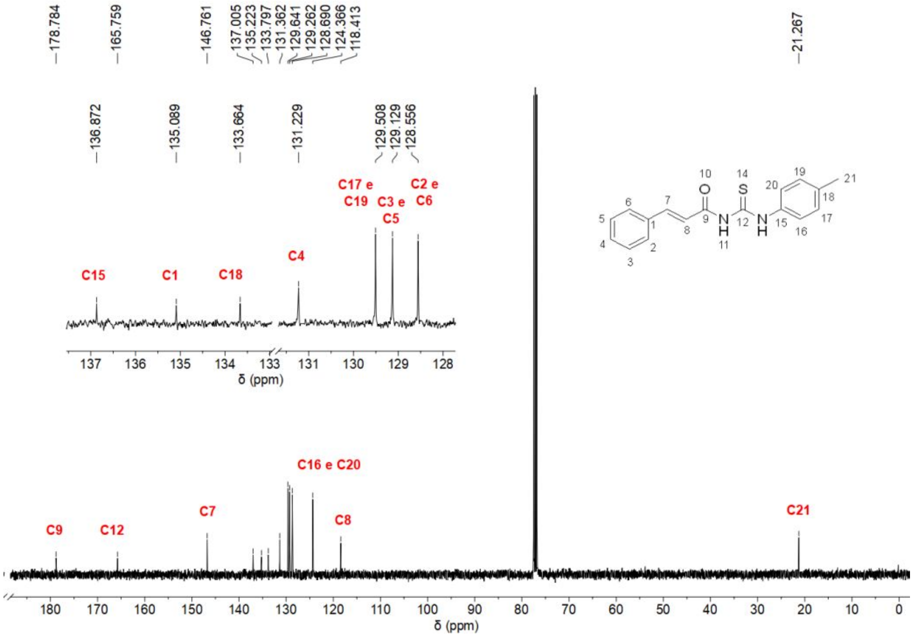

Figure S9 – ^{13}C NMR spectrum (100 MHz, $CDCl_3$) of **LMed 46**

***N*-(*p*-tolylcarbamothioyl)cinnamamide (LMed 46):** Yellow solid; 90% yield. MP: 198-199 ºC **Lit:** 183-186 ºC. NMR ^{1}H [$CDCl_3$, 400 MHz] δ: 12.52 (s, 1H), 9.03 (s, 1H), 7.86 (d, J = 15.5 Hz, 1H), 7.63 – 7.53 (m, 4H), 7.51 – 7.40 (m, 3H), 7.27 – 7.19 (m, 2H), 6.54 (d, J = 14.9 Hz, 1H), 2.39 (s, 3H). NMR ^{13}C [$CDCl_3$, 100 MHz] δ: 178.78, 165.76, 146.76, 137.01, 135.22, 133.80, 131.36, 129.64, 129.26, 128.69, 124.37, 118.41, 21.27.

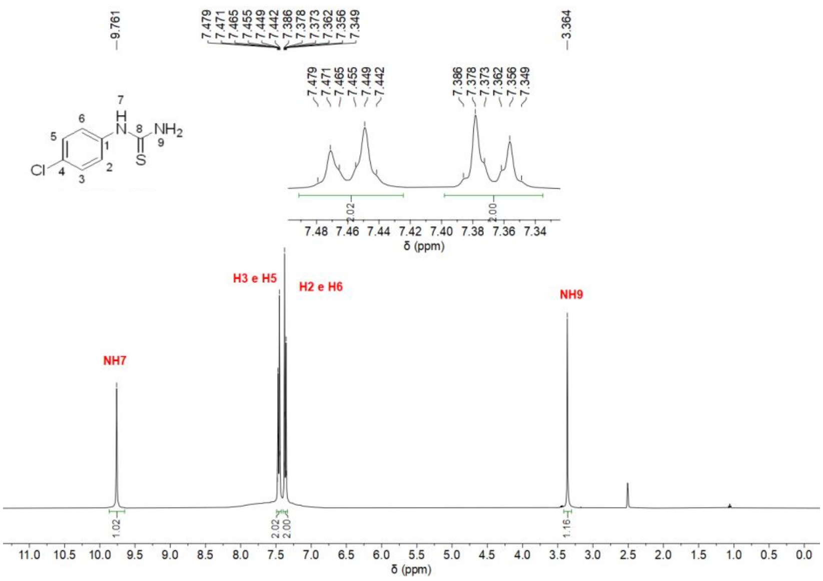

Figure S10 – ^{1}H NMR spectrum (400 MHz, DMSO-d_6) of **LMed 122**

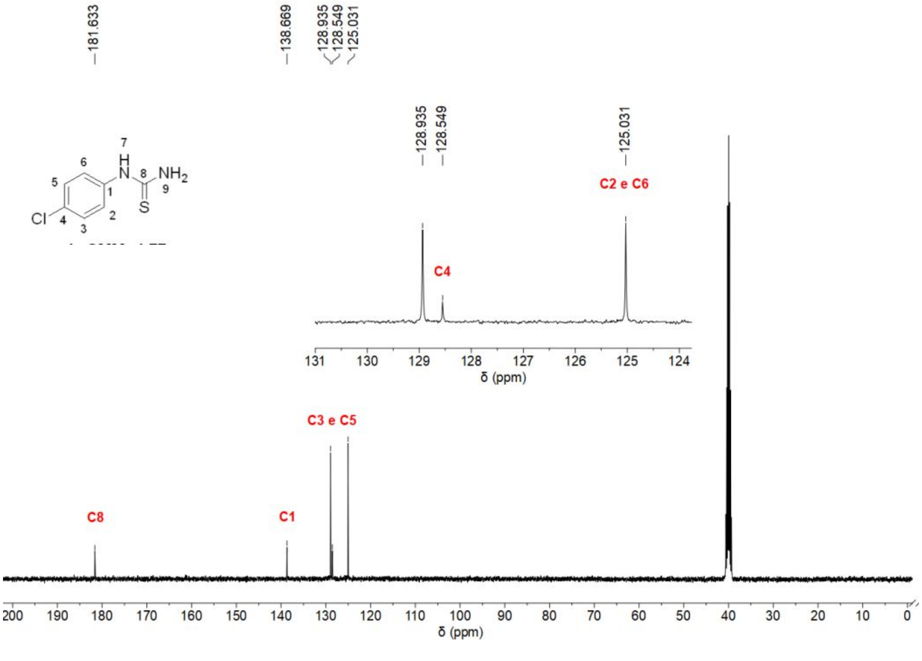

Figure S11 – ^{13}C NMR spectrum (100 MHz, DMSO-d_6) of **LMed 122**

1-(4-chlorophenyl)thiourea (LMed 122): White crystal; 35% yield. MP: 171-172 ºC. **Lit**: 172 ºC (HAY *et al.*, 2010) . NMR ^{1}H [DMSO-d_6, 400 MHz] δ: 9.76 (s, 1H), 7.49 – 7.42 (m, 2H), 7.40 – 7.34 (m, 2H), 3.36 (s, 1H). NMR ^{13}C [DMSO-d_6, 100 MHz] δ: 181.63, 138.67, 128.94, 128.55, 125.03.

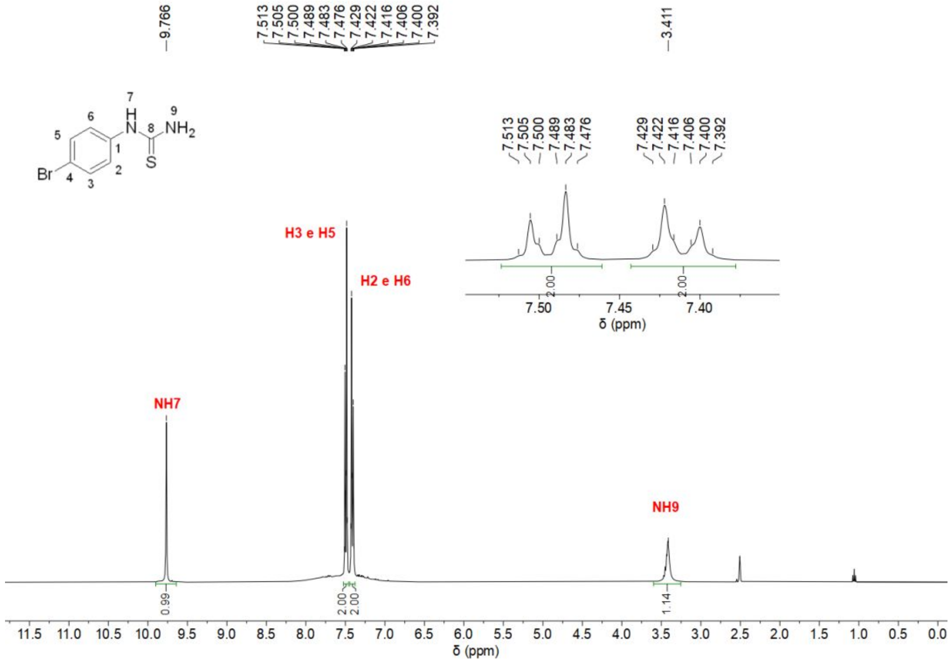

Figure S12 – ^{1}H NMR spectrum (400 MHz, DMSO-d_6) of **LMed 123**

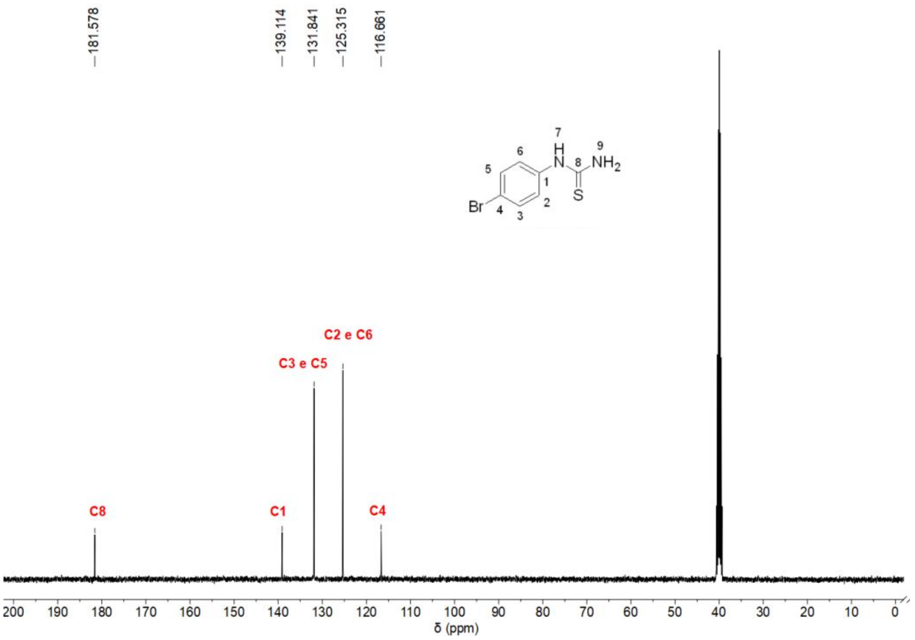

Figure S13 – ^{13}C NMR spectrum (100 MHz, DMSO-d_6) of **LMed 123**

1-(4-Bromophenyl)thiourea (LMed 123): Yellow crystal; 50% yield. MP: 172-173 ºC. **Lit**: 171 ºC (GUPTA *et al.*, 2010). NMR ^{1}H [DMSO-d_6, 400 MHz] δ: 9.77 (s, 1H), 7.52 – 7.46 (m, 2H), 7.44 – 7.38 (m, 2H), 3.41 (s, 1H). NMR ^{13}C [DMSO-d_6, 100 MHz] δ: 181.58, 139.11, 131.84, 125.32, 116.66.

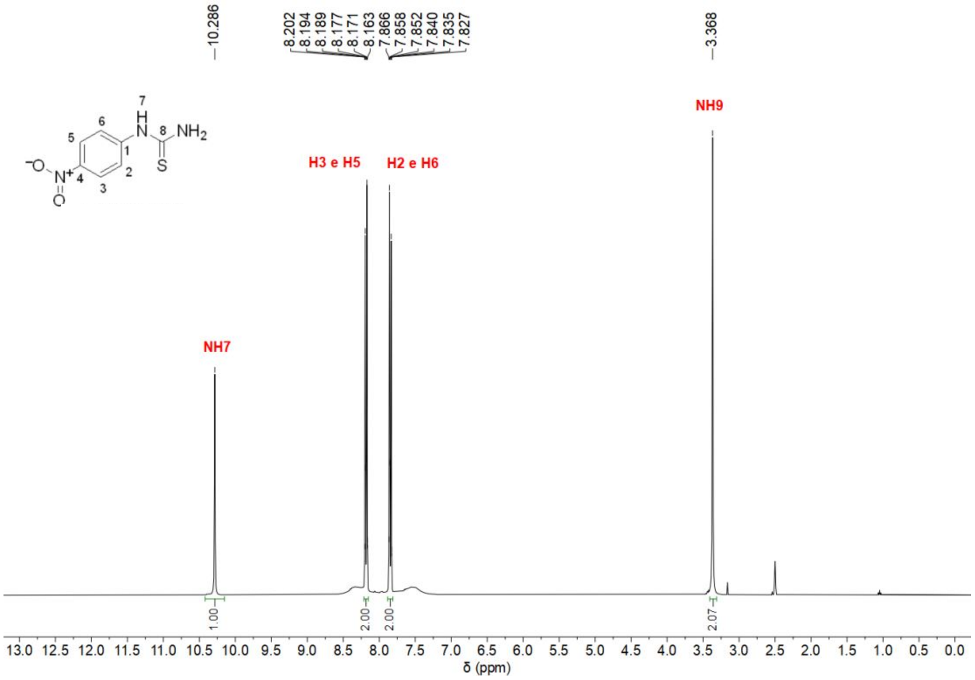

Figure S14 – ^{1}H NMR spectrum (400 MHz, DMSO-d_6) of **LMed 124**

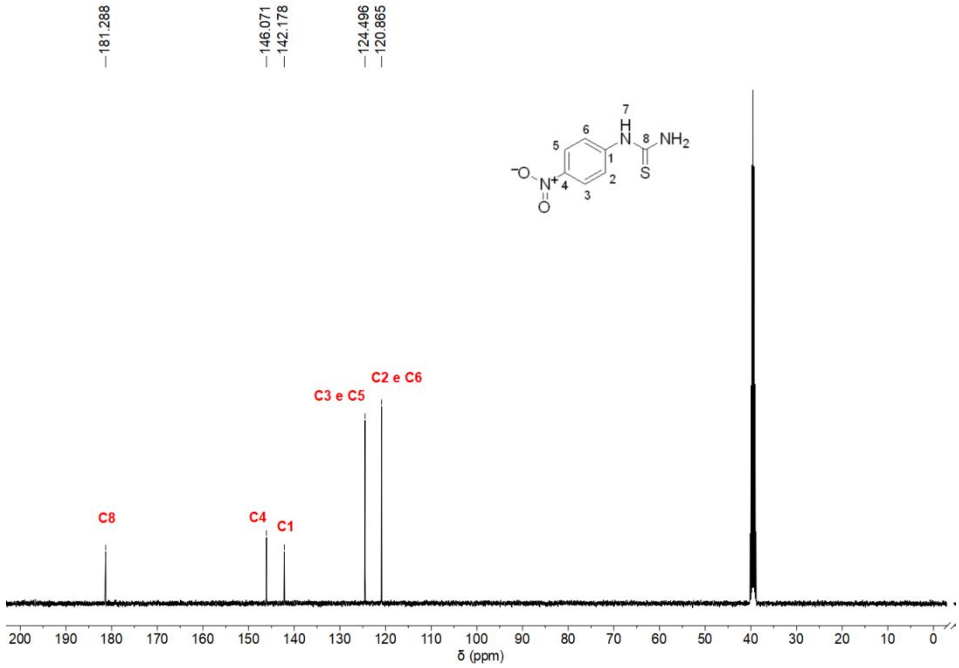

Figure S15 – ^{13}C NMR spectrum (100 MHz, DMSO-d_6) of **LMed 124**

1-(4-Nitrophenyl)thiourea (LMed 124): Brown solid; 38% yield. MP: 197-198 ºC. **Lit**: 198 ºC. NMR ^{1}H [DMSO-d_6, 400 MHz] δ: 10.29 (s, 1H), 8.21 – 8.15 (m, 2H), 7.88 – 7.81 (m, 2H), 3.37 (s, 2H). NMR ^{13}C [DMSO-d_6, 100 MHz] δ: 181.29, 146.07, 142.18, 124.50, 120.87.

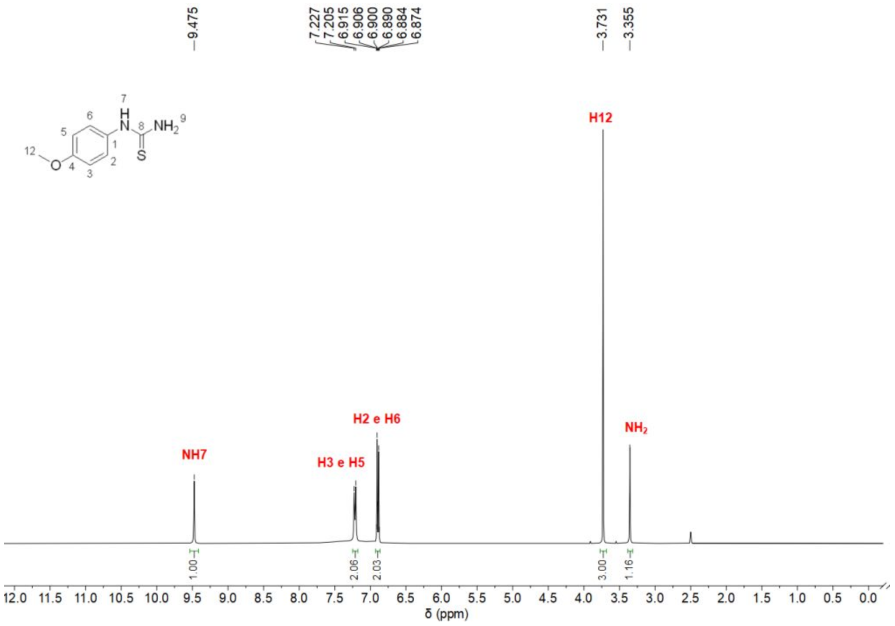

Figure S16 – ^{1}H NMR spectrum (400 MHz, DMSO-d_6) of **LMed 125**

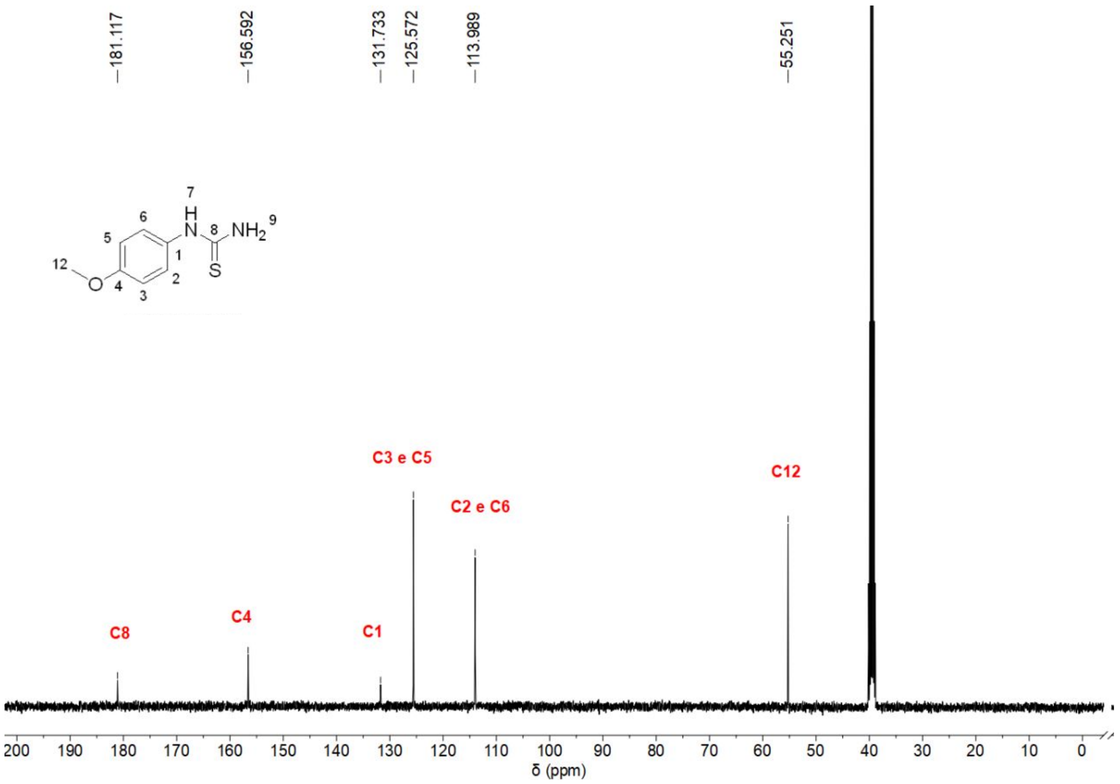

Figure S17 – ^{13}C NMR spectrum (100 MHz, DMSO-d_6) of **LMed 125**

1-(4-methoxyphenyl)thiourea (LMed 125): Gray solid; 48% yield. MP: 211-212 ºC. **Lit**: 209-210 ºC. NMR ^{1}H [DMSO-d_6, 400 MHz] δ: 9.48 (s, 1H), 7.22 (d, J = 8.9 Hz, 2H), 6.93 – 6.87 (m, 2H), 3.73 (s, 3H), 3.36 (s, 1H). NMR ^{13}C [DMSO-d_6, 100 MHz] δ: 181.12, 156.59, 131.73, 125.57, 113.99, 55.25.

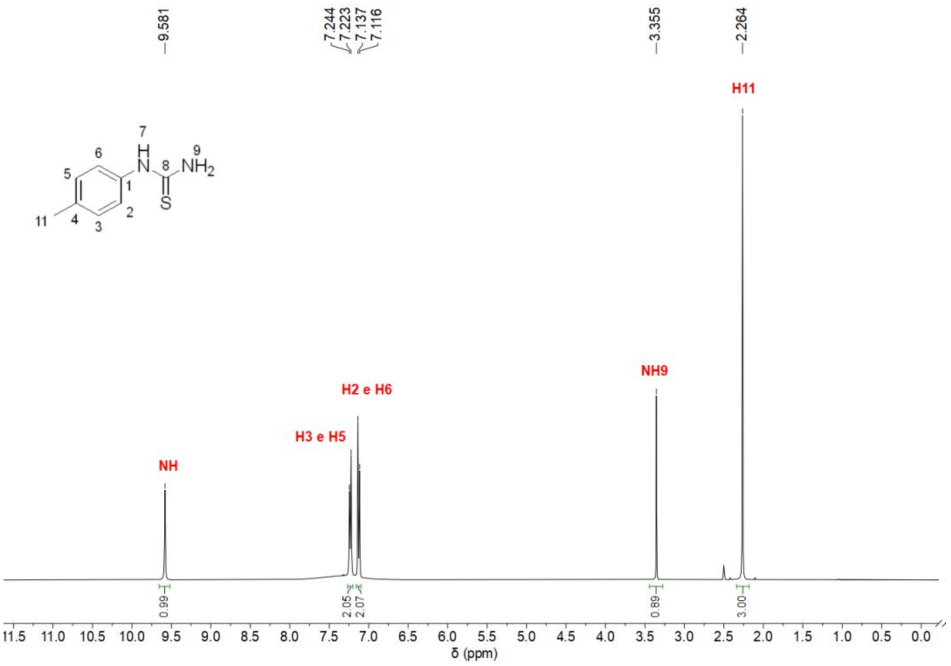

Figure S18 – ^{1}H NMR spectrum (400 MHz, DMSO-d$_6$) of **LMed 126**

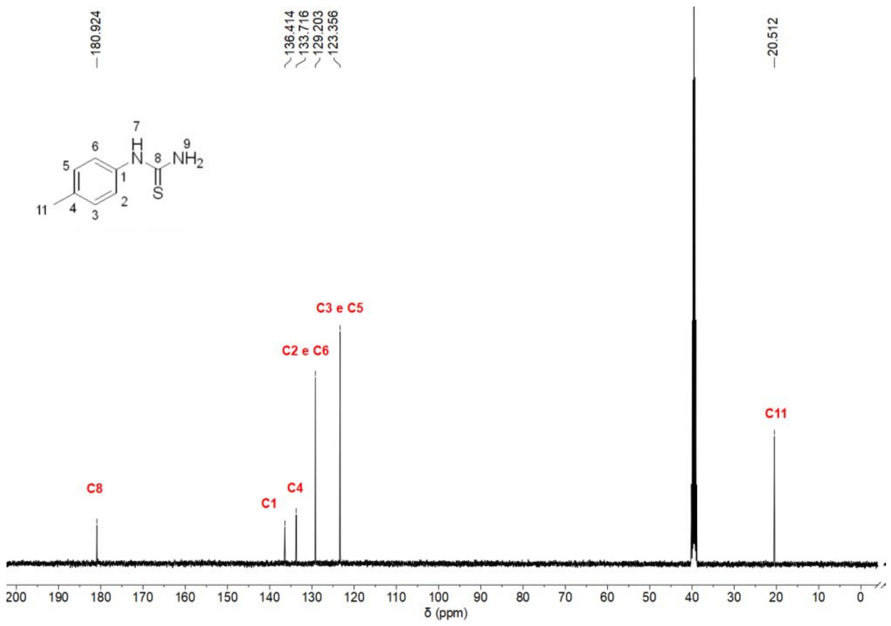

Figure S19 – ^{13}C NMR spectrum (100 MHz, DMSO-d$_6$) of **LMed 126**

1-(*p*-tolyl)thiourea (LMed 126): White solid; 46% yield. MP: 189-191 ºC. **Lit**: 190 ºC. NMR ^{1}H [DMSO-d$_6$, 400 MHz] δ: 9.58 (s, 1H), 7.23 (d, J = 8.4 Hz, 2H), 7.13 (d, J = 8.4 Hz, 2H), 3.35 (s, 1H), 2.26 (s, 3H). NMR ^{13}C [DMSO-d$_6$, 100 MHz] δ: 180.92, 136.41, 133.72, 129.20, 123.36, 20.51.
